# The dynamin-related protein PfDyn2 is essential for both apicoplast and mitochondrial fission in *Plasmodium falciparum*

**DOI:** 10.1128/mbio.03036-24

**Published:** 2024-11-29

**Authors:** Alexander A. Morano, Wei Xu, Francesca M. Navarro, Neeta Shadija, Jeffrey D. Dvorin, Hangjun Ke

**Affiliations:** 1 Division of Infectious Diseases, Boston Children’s Hospital, Boston, Massachusetts, USA; 2 Biological and Biomedical Sciences, Harvard Medical School, Boston, Massachusetts, USA; 3 Center for Molecular Parasitology, Department of Microbiology and Immunology, Drexel University College of Medicine, Philadelphia, Pennsylvania, USA; 4 Department of Pediatrics, Harvard Medical School, Boston, Massachusetts, USA; Rutgers-New Jersey Medical School, Newark, New Jersey, USA

**Keywords:** malaria, *Plasmodium falciparum*, dynamin, dynamin-related protein, PfDyn2, apicoplast fission, mitochondrial fission, apicoplast, mitochondria, basal complex, schizogony, residual body

## Abstract

Dynamins, or dynamin-related proteins (DRPs), are large mechano-sensitive GTPases that mediate membrane dynamics or organellar fission/fusion events. *Plasmodium falciparum* encodes three dynamin-like proteins whose functions are poorly understood. Here, we demonstrate that one of these dynamin-related proteins, PfDyn2, is required to divide both the apicoplast and the mitochondrion, a striking divergence from the biology of related parasites. Using super-resolution and ultrastructure expansion microscopy (U-ExM), we show that PfDyn2 is expressed in dividing schizonts, and that it localizes to both the apicoplast and the mitochondrion. Our use of long-term, live-cell microscopy allows for the visualization of apicoplast and mitochondrial division in live parasites at super resolution for the first time, and demonstrates that in PfDyn2-deficient parasites, while the apicoplast and mitochondrion increase in size and complexity, they do not undergo fission. We also show that these organellar fission defects prevent successful individualization of the schizont mass and the formation of new daughter cells, or merozoites because the basal complex, the cytokinetic ring of *Plasmodium*, cannot fully contract in PfDyn2-deficient parasites, a phenotype secondary to physical blockage by undivided organelles occluding the ring. PfDyn2’s singular role in mediating both apicoplast and mitochondrial fission has not been observed in other organisms possessing two endosymbiotic organelles, including other Apicomplexans, thus reflecting a unique, potentially exploitable method of organellar division in *P. falciparum*.

**IMPORTANCE:**

*Plasmodium falciparum* remains a significant global pathogen, causing over 200 million infections and over 600,000 deaths per year. One significant obstacle to the control of malaria is increasing resistance to first-line artemisinin-based antimalarials. Another is a lack of basic knowledge about the cell biology of the parasite. Along with the mitochondrion, *Plasmodium* contains a second organelle descended from an endosymbiotic event, the apicoplast. Both organelles are common targets for antimalarials, but because many proteins involved in organellar fission are not conserved in *Plasmodium*, until now, the mechanisms underlying apicoplast and mitochondrial division have been unknown. In this study, we demonstrate that PfDyn2, a dynamin-related protein (DRP), is required for the division of both organelles. We also show that defects in organellar division hinder segmentation of the schizont and formation of invasive merozoites by preventing full contraction of the basal complex. By demonstrating its necessity for the proper division of both the apicoplast and the mitochondria, this study highlights PfDyn2 as a potential target for new antimalarials.

## INTRODUCTION

Malaria remains a significant global health burden, with 249 million cases and 608,000 deaths in 2022 (1). *Plasmodium falciparum* is the causative agent of the most dangerous form of malaria, and it is the asexual replication of *Plasmodium* parasites within human red blood cells that causes the characteristic cyclical fevers of malaria as well as the potentially lethal complications including severe anemia, renal failure, and neurologic complications (cerebral malaria).

*P. falciparum* harbors two distinct bacterially derived organelles, a single mitochondrion and a single apicoplast. The mitochondrion, tracing its origin to an alpha-protobacterium akin to other eukaryotes, is a key target for antimalarial drugs (2–4). Clinical interventions such as atovaquone (5–7), introduced in 2000, and a multitude of inhibitors undergoing pre-clinical or clinical trials (8–12) disrupt the parasite’s mitochondrial electron transport chain or ability to synthesize pyrimidines. The apicoplast is a unique four-membrane-surrounded structure originating from a cyanobacterium through primary and secondary endosymbiosis (13–16). It has been the target of compounds used to treat malaria since the 1950s (17) and continues to be an important target for developing novel antimalarials (18–22). These two organelles house numerous biochemical pathways, such as synthesis of pyrimidines (23), iron-sulfur clusters (24), adenosine triphosphate (ATP) (25), heme (26), lipids (27), isoprenoid precursors (28), and coenzyme A (CoA) (29). These metabolites are required for parasites to progress through their life cycle in both human and mosquito hosts. Despite the wealth of knowledge about their biochemical functions and significance for drug development, the mechanisms underlying the division of these organelles remain unknown. During the 48-hour asexual blood stage (ABS), the mitochondrion and apicoplast transform from small globular structures to large tubular networks in dividing parasites (30–32). At the end of schizogony (33, 34), the specialized cytokinesis of *Plasmodium* and related parasites, the branched mitochondrion and apicoplast are divided into 16–32 copies and subsequently distributed into all progeny, ensuring that each daughter cell (merozoite) acquires one copy of each organelle (35, 36). Because the mitochondrion and apicoplast cannot be made *de novo*, organellar expansion and fission processes are prerequisites for generating functional merozoites. A recent focused ion beam-scanning electron microscopy study hypothesizes that interaction of the apicoplast with the centriolar plaque is important for this one-to-one organellar segregation (36). Despite this innovative observation, no molecular mechanisms for this association have been identified. Additionally, because *P. falciparum* does not encode FtsZ or other proteins present in the bacterial fission machinery (2), and the protein annotated as mitochondrial fission protein 1 (Fis1) is dispensable (37), the parasite must rely on unique organellar fission mechanisms.

Dynamins, or dynamin-related proteins (DRPs), mediate membrane scission or fusion events across various cellular processes, including endocytosis, organelle division, and intracellular trafficking (38). These large GTPases form helical structures around the membranes through self-oligomerization, generating constrictive force to divide their cellular targets (39). The role of dynamins and DRPs in mitochondrial fission has been extensively studied in model organisms: Drp1 has long been known to function in mitochondrial fission, but recent research has revealed that it is not the sole actor responsible for this process (40–42). Actin fibers initially mark “fission sites,” preconstructed regions of the mitochondrion, to which Drp1 is recruited. Drp1 then assembles into helical structures, wherein its GTPase activity forces further constriction, although not yet true fission, of the dividing organelle (40, 41). Dyn2 is then responsible for the final fissive act, although the coordination of their activities remains unclear. It is known, however, that Dyn2’s proline-rich domain is required for recruitment to the mitochondrial membrane, and that both dynamin-related proteins are capable of binding actin filaments to generate sufficient force during membrane fission (43–46). In addition, mitochondrial adaptor proteins are required for catalysis of fission, likely by enabling interaction between the membrane surface and the DRP in question. The co-assembly of mitochondrial adaptor proteins with DRPs reduces significantly the diameter of the helical structures they form, emphasizing the necessity of these adaptors for mitochondrial fission (47). The pleckstrin homology domain that allows for interaction with phospholipids in other dynamins, including in Dyn2, is absent in Drp1; thus, its vital role in mitochondrial fission is necessarily mediated by one, if not many, adaptor proteins (48).

Apicomplexan parasites, including *Plasmodium* spp., contain three phylogenetically distinct dynamin-related proteins (49–52). In the related apicomplexan parasite *Toxoplasma gondii,* TgDrpA, is required for apicoplast fission but dispensable for mitochondrial fission (49). Previous studies indicate that PfDyn1 (PF3D7_1145400) and PfDyn2 (PF3D7_1037500) possess GTPase activity (53), and suggest that PfDyn2 localizes to the endoplasmic reticulum (ER), Golgi, and apicoplast, but the precise localization as well as the function of these proteins remains elusive (54, 55).

In this study, employing multiple transgenic parasite strains, super-resolution and ultrastructure expansion microscopy (U-ExM), and long-term, live-cell super-resolution microscopy, we discover that PfDyn2 localizes to both the apicoplast and the mitochondrion in schizont-stage parasites and is required for the division of both organelles. Conditional depletion of PfDyn2 prevents organellar fission, causing additional, profound defects in the formation of viable merozoites from the schizont mass. Our findings illuminate a unique aspect of *P. falciparum* biology—in no other organism that possesses two bacterially derived organelles does a single DRP govern both organelles’ division. Thus, PfDyn2’s dual role also identifies it as a potentially valuable target for novel antimalarials.

## RESULTS

### PfDyn2 localizes to the apicoplast and mitochondrion, and is essential for asexual stage development

The *P. falciparum* genome encodes three dynamin-related proteins, PfDyn1 (PF3D7_1145400), PfDyn2 (PF3D7_1037500), and PfDyn3 (PF3D7_1218500) (Fig. S1A). Previous phylogenetic analyses determined that apicomplexan DRPs form distinct evolutionary clades diverging from other dynamins, including ARC5, the dynamin involved in plastid division in plants and red alga (49, 50). Apicomplexan dynamins lack a bioinformatically identifiable lipid-binding Pleckstrin-Homology (PH) (56), and, thus, likely require additional adaptor proteins to bind to membranes of the organelles they work to divide (48). PfDyn2 was selected for study because it is the most similar to *T. gondii* TgDrpA among *P. falciparum* dynamin-related proteins (Fig. S1B). To allow for both localization and translational control of PfDyn2, we endogenously fused three copies of the hemagglutinin (3HA) epitope to PfDyn2’s C-terminus and produced the parasite line NF54attB-PfDyn2-3HA^apt^ (PfDyn2-3HA^apt^) (Fig. S1C). As described previously (57, 58), the TetR-DOZI-aptamer system regulates gene expression using a small molecule, anhydrotetracycline (aTc); the target protein is translated in the presence of aTc and is knocked down in its absence. The PfDyn2-3HA^apt^ genotype was confirmed by PCR (Fig. S1D), and the tagged protein was expressed at the expected molecular weight (Fig. S1E).

**Fig 1:**
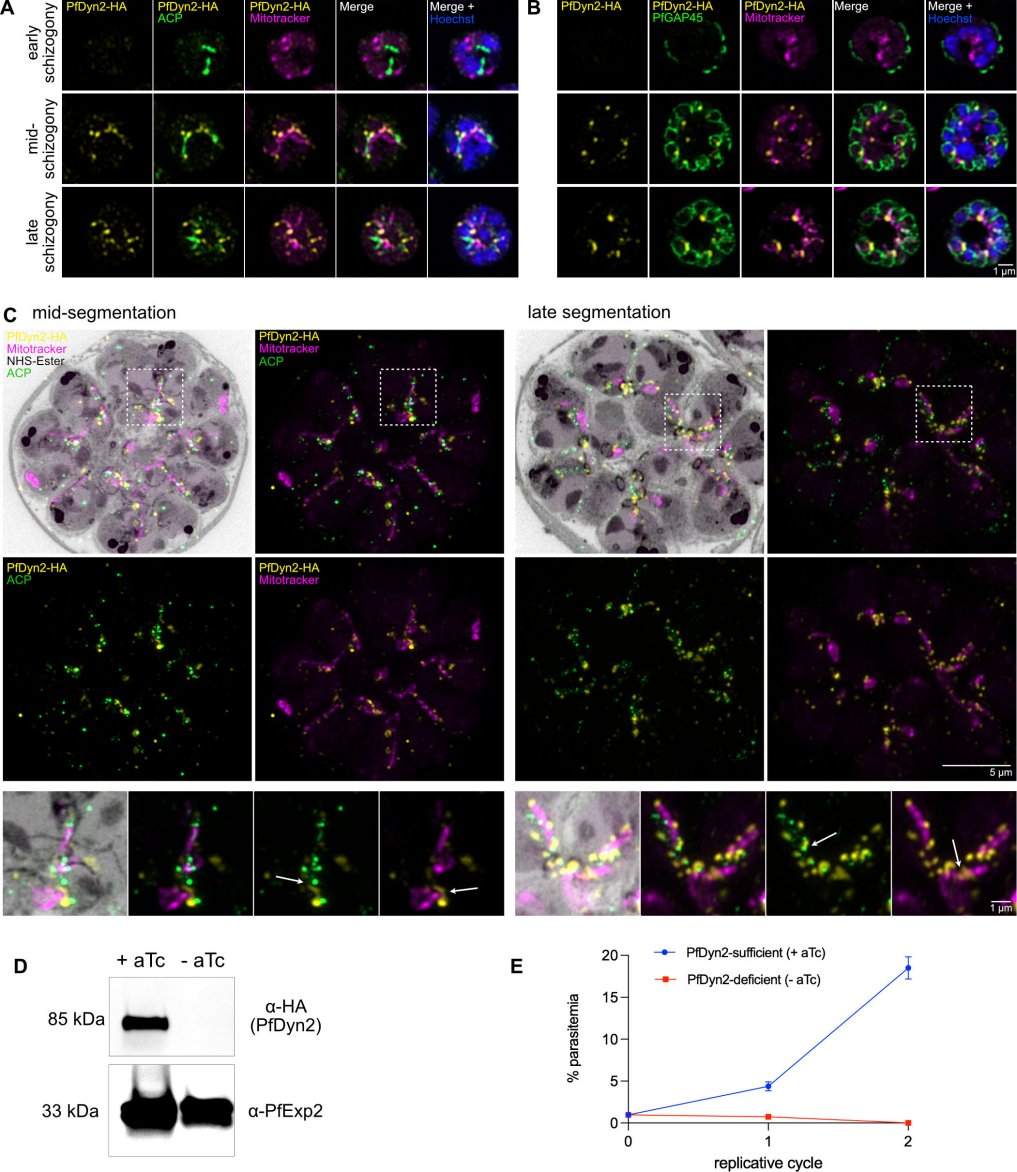
PfDyn2 localizes to the apicoplast and mitochondria, and is essential for growth and replication. (**A**) Super-resolution microscopy showing PfDyn2’s localization to the apicoplast and mitochondria at different stages of schizogony in PfDyn2-3HA^apt^ parasites. PfDyn2 was probed with anti-HA. The apicoplast was probed with α-PyACP (acyl carrier protein). Mitochondria were labeled with MitoTracker Orange CMTMRos. Nuclei were stained by Hoechst. (**B**) Super-resolution microscopy verifies PfDyn2’s localization to mitochondria at different stages of IMC development in the PfDyn2-3HA^apt^ parasites. IMC was probed by α-PfGAP45. PfDyn2, mitochondria, and nuclei were labeled as shown in (**A**). (**C**) U-ExM of PfDyn2-3HA^apt^ parasites showing localization of PfDyn2 in mid- and late schizogony. PfDyn2, the apicoplast, and mitochondria were labeled as shown in (**A**). Proteins were labeled by AlexaFluor 405 NHS-Ester and shown in gray. White boxes indicate regions zoomed-in below the larger images; white arrows highlight PfDyn2 encircling the branches of the apicoplast or mitochondrion. (**D**) Immunoblot showing extent of PfDyn2 knockdown over 36 hours following aTc removal. PfExp2, exported protein 2, was used as a loading control. (**E**) Growth curve comparing replicative fitness of PfDyn2-sufficient (+aTc) and -deficient (–aTc) parasites over 96 hours (two replicative cycles). Data shown as mean ± SD (*n* = 3). All scale bars = 1 µm, except for the non-inset images in (C) where scale bars = 5 µm.

To evaluate PfDyn2’s subcellular localization, we performed immunofluorescence assays (IFAs). PfDyn2 is expressed in schizont-stage but not in ring- or trophozoite-stage parasites (Fig. S2), as previously reported (55). Using super-resolution microscopy, in early schizogony, when there are fewer than five nuclei and the inner membrane complex (IMC) is first visible, PfDyn2 is undetectable (Fig. 1A and B). During middle and late schizogony, PfDyn2 localizes to the apicoplast and the mitochondrion, with increased intensity at the ends of the organelles and nascent “branch points”, where separation will later occur (Fig. 1A and B). To verify PfDyn2’s organellar localization with improved spatial resolution, we performed U-ExM (59). Via U-ExM, in middle and late segmentation, PfDyn2 still localized to both organelles, appearing as dots along the lengths of the apicoplast and mitochondria, as well as rings surrounding them (Fig. 1C). In addition, there are occasionally small foci of PfDyn2 staining that are present in regions not immediately adjacent to the apicoplast or mitochondria. These likely represent background staining, especially in U-ExM images where background is often higher.

To assess PfDyn2’s essentiality, we compared replication rates of PfDyn2-deficient and -sufficient parasites. Following aTc removal, PfDyn2 protein falls below the detection limit after approximately 36 hours (Fig. 1D), and parasitemia of the PfDyn2-deficient (–aTc) culture fails to increase (Fig. 1E), establishing that PfDyn2 is essential for asexual growth and replication. To better understand the knockdown phenotype, we performed a detailed time course experiment (Fig. S3). In a tightly synchronized ring-stage culture (~8 hours post-invasion, hpi), we initiated knockdown and examined parasite morphology and PfDyn2 protein levels at specific time points. PfDyn2-deficient parasites first appear morphologically abnormal at the mid-schizont stage (Fig. S3A). While PfDyn2-sufficient parasites (+aTc) complete segmentation, egress, and reinvasion, PfDyn2-deficient parasites (–aTc) arrest as late schizonts and die shortly thereafter. Immunoblot analysis showed that PfDyn2 (+aTc) is not expressed until approximately 40 hpi (Fig. S3B), corroborating our immunofluorescence data (Fig. 1; Fig. S2). These results demonstrate that PfDyn2 is essential for schizont-stage development.

Previous studies suggest that the primary role of the apicoplast during the ABS is to synthesize isoprenoids (28). Indeed, genetic knockdown of many essential apicoplast genes can be rescued by exogenous isopentenyl pyrophosphate (IPP, 200 µM) (24, 60–70). We thus attempted to rescue PfDyn2-deficient parasites with IPP. We initiated PfDyn2 knockdown for 36 hours then added IPP (200 µM) or aTc and monitored parasite growth for three additional days. We found that exogenous IPP does not rescue PfDyn2 deficiency; IPP-treated parasites resembled the –aTc counterparts (Fig. S4A and B). To ensure our IPP was functional, we performed growth inhibition assays with fosmidomycin (60), an apicoplast inhibitor that is detoxified by IPP. Parasites (NF54attB, wild type) were sensitive to fosmidomycin as expected (EC_50_ = 1.12 µM), but IPP fully restored their growth under drug treatment (Fig. S4C). The defect in PfDyn2-deficient parasites thus cannot be rescued with isoprenoid supplementation.

### PfDyn2 is required for apicoplast fission

Using the attB/attP integration system (71), we inserted ACP_L_-mRuby into the genome of PfDyn2-3HA^apt^ parasites. The leader sequence of ACP (ACP_L_), the first 55 amino acids of acyl carrier protein, has been shown to guide fluorescent proteins into the apicoplast matrix (30, 72). In the PfDyn2-3HA^apt^-ACP_L_-mRuby parasite line, we first verified apicoplast morphologies throughout the asexual blood stage (Fig. S5). In agreement with previous work (30), we observed that the apicoplast begins elongating and branching to form a network at around 30 hpi. Between segmentation and egress, apicoplasts are then distributed into individual merozoites.

**Fig 2:**
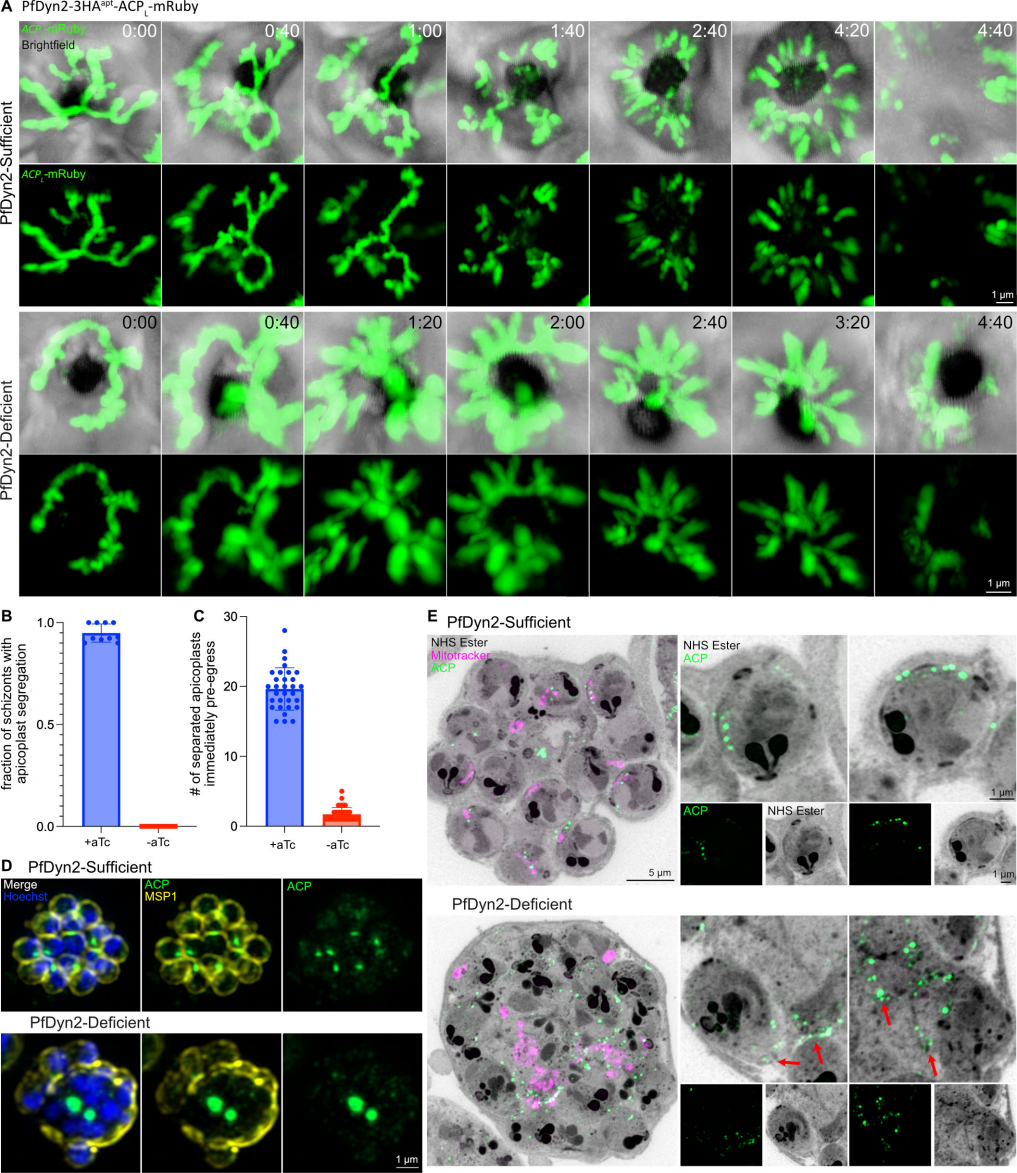
PfDyn2 mediates apicoplast fission. (**A**) Images from live-cell video microscopy demonstrating apicoplast division in the PfDyn2-3HA^apt^-ACP_L_-mRuby parasites. The top two rows include images from Video S1 (PfDyn2 sufficient) and the bottom two rows include images from Video S2 (PfDyn2 deficient). Time is displayed as hours:minutes. (**B**) Quantification of PfDyn2-3HA^apt^-ACP_L_-mRuby parasites that demonstrated apicoplast segmentation in PfDyn2-sufficient and -deficient conditions under live-cell video microscopy. Data shown as mean ± SD with individual values overlaid on bar. (**C**) Quantification of individual separated apicoplasts present immediately pre-egress in PfDyn2-sufficient and -deficient conditions during live-cell video microscopy. Data shown as mean ± SD with individual values overlaid on bar. (**D**) Super-resolution microscopy comparing apicoplast division in PfDyn2-sufficient and -deficient conditions. Late schizont-stage parasites were treated with E64 to block egress prior to fixation. PfMSP1(α-PfMSP1) is in yellow, the apicoplast (α-PyACP [acyl carrier protein]) is in green, and nuclei were stained by Hoechst. (**E**) U-ExM of PfDyn2-3HA^apt^ parasites comparing apicoplast division in PfDyn2-sufficient and -deficient conditions. Late schizont-stage parasites were treated with E64 to block egress prior to fixation. Mitochondria (MitoTracker Orange CMTMRos) are in magenta, apicoplast (α-PyACP) is in green. AF405 NHS Ester was used as a general protein stain and is shown in gray. Maximum projections of approximately 40 z-slices are shown on the left. To the right of each large image are zoomed-in views of selected nascent merozoites (shown as maximum projections of 5–10 z-slices) comparing apicoplast segmentation in PfDyn2-sufficient and -deficient schizonts. For the zoomed-in images, the selected merozoites were selected from a subset of z-slices from the larger image. We note that the zoomed-in panels show some overlap of incompletely separated merozoites for the PfDyn2-deficient parasites. Red arrows point to apicoplasts attached to or leading out from the basal complex. All scale bars = 1 µm except for larger U-ExM images where the scale bar = 5 µm. A–D, data shown are derived from two biological replicates.

Using super-resolution, long-term live-cell microscopy, we then tracked PfDyn2-sufficient and -deficient parasites. In PfDyn2-sufficient parasites, we observed sequential apicoplast fission over the course of segmentation (Fig. 2A; Video S1). The apicoplast first formed a branching structure (Fig. 2A, top row, 0:40). Next, branches were separated (Fig. 2A, top row, 1:00–1:40), then individual apicoplasts were separated as they were packaged into merozoites (Fig. 2A, top row, 2:40–4:20). Immediately before egress (pre-egress) (Fig. 2A, top row, 4:20), a clear separation between apicoplasts was evident with 22 distinct apicoplast structures. PfDyn2-deficient parasites were still able to form branched apicoplasts (Fig. 2A, bottom row, 0:40–1:20), but only a single division resulting in two separated, multi-branched structures was made during segmentation (Fig. 2A, bottom row, 2:40–3:20; Video S2). In the absence of PfDyn2, only one of the branch cuts was made and no individual apicoplasts were formed, indicating that PfDyn2 is required for the earliest stages of apicoplast fission. Upon egress, the still-connected apicoplasts of PfDyn2-deficient parasites collapsed within the residual body (Fig. 2A, bottom row, 4:40). To quantify this phenotype, we first examined >100 parasites per condition, which progressed to egress during long-term live-cell imaging. PfDyn2-sufficient parasites showed obvious apicoplast segregation immediately pre- or post-egress (95% ± 4.5% per spatial position), whereas no PfDyn2-deficient parasites displayed clear apicoplast segregation (0% ± 0% per spatial position) (Fig. 2B). For this quantification, clear apicoplast segregation was counted as present if there was an apicoplast inside of a fully segmented merozoite. The application of super-resolution microscopy to long-term, live-cell experiments allowed us to perform further quantification to determine when in the process of apicoplast fission PfDyn2 is required by counting the number of individual breaks in the apicoplasts present in selected parasites immediately pre-egress. In PfDyn2-sufficient parasites, the mean number of apicoplasts visible was 20 ± 3, with variation in the number of apicoplasts counted based on the z-position and angle of each parasite at the pre-egress time point as well as the inherent variation in the number of merozoites produced by each schizont (Fig. 2C). In contrast, PfDyn2-deficient parasites only had 2 ± 1 visibly separated apicoplast-like structures (as they were not individuated apicoplasts in this condition) present immediately pre-egress. These fission events (or breaks) revealed that there were either some subsegmental fission events occurred or regions of apicoplast that were too thin to be visualized by the fluorescent marker. Interestingly, the majority (56%) of PfDyn2-deficient parasites examined proceeded to egress without any fission events between any branches of the apicoplast structure. Only 18% of the parasites examined had undergone more than one fission event, and zero fully segmented schizont contained a separate and segregated individual apicoplast in each merozoite.

We then examined apicoplast fission in PfDyn2-sufficient and -deficient conditions with greater spatial resolution in fixed cells. We treated parasites with E64 prior to fixation, a cysteine-protease inhibitor that prevents rupture of the RBC plasma membrane (73), to enrich late schizont-stage parasites that have normally finished organellar fission. Using super-resolution immunofluorescence, we labeled the apicoplast with an ACP antibody and the parasite plasma membrane with an antibody against PfMSP1, merozoite surface protein 1 (74). The proper distribution of PfMSP1 to the merozoite surface serves as a marker for normal segmentation. PfDyn2-sufficent schizonts form evenly sized and shaped merozoites, each with a copy of the apicoplast; however, PfDyn2-deficient parasites have a large central mass of apicoplast material with the plasma membrane surrounding multiple nuclei in this structure indicative of segmentation failure (Fig. 2D). Using U-ExM and markers for the apicoplast (ACP antibody) and mitochondria (MitoTracker) simultaneously, we observed that, in PfDyn2-sufficient parasites, the apicoplast successfully separated in individual merozoites (Fig. 2E). However, in PfDyn2-deficient parasites, the apicoplast showed a lack of division, again with a large central mass, and undivided apicoplast branches connected merozoites through the basal complex (Fig. 2E, red arrows). Together, these results strongly indicate that PfDyn2 mediates apicoplast fission, and indeed that PfDyn2 is required for the earliest steps of apicoplast fission to be completed effectively.

### PfDyn2 is required for mitochondrial fission

We then inserted Strep II-mNeonGreen-Tom22 into the genome of PfDyn2-3HA^apt^ parasites using the attB/attP system, generating the parasite line PfDyn2-3HA^apt^-StrepII-mNeonGreen-Tom22 (PfDyn2-3HA^apt^-mNG-Tom22 in short). In agreement with the prior study (30), the mitochondrion displayed the expected developmental progression from a globular structure to a branched tubular network over the course of asexual development (Fig. S6).

**Fig 3:**
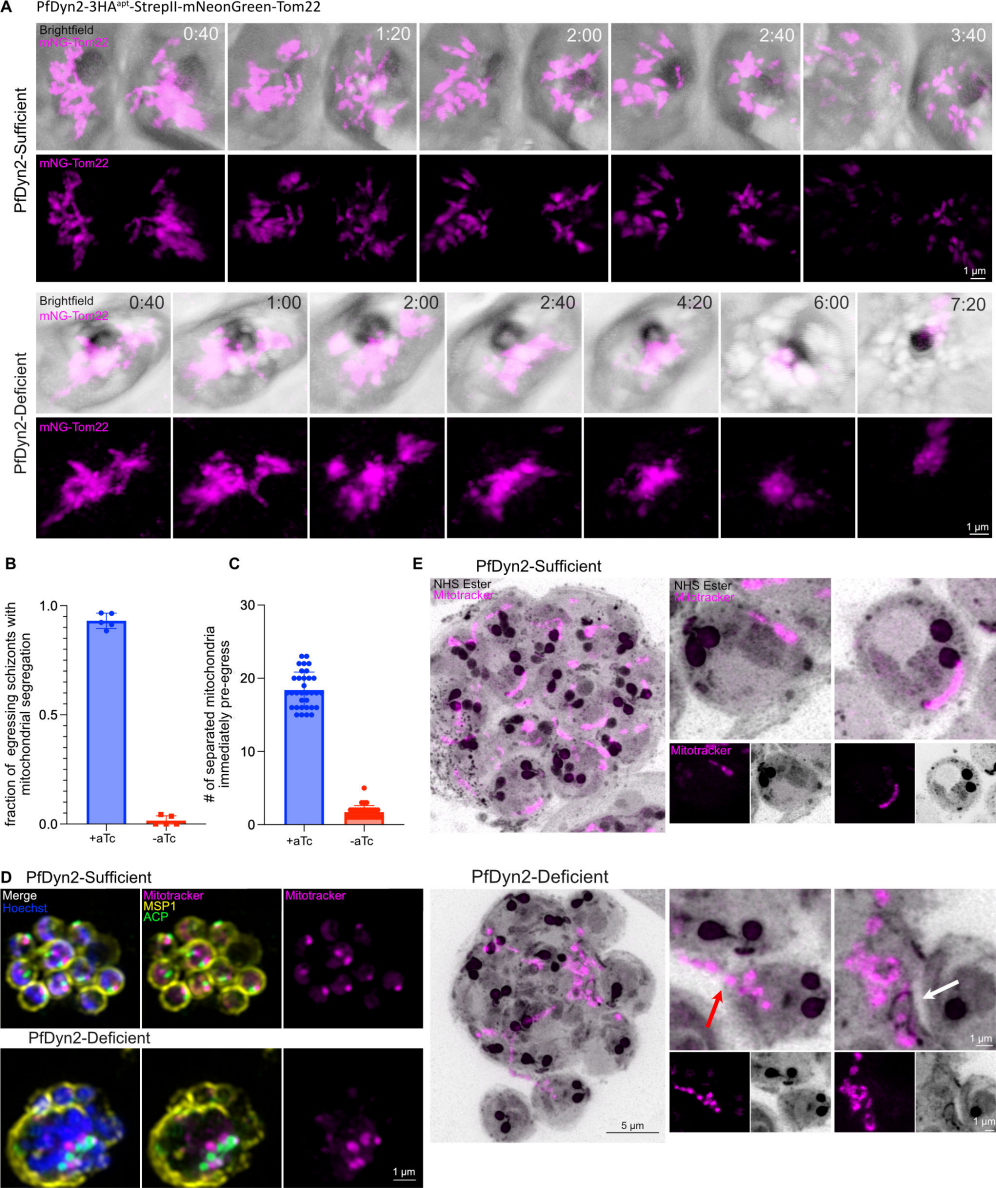
PfDyn2 mediates mitochondrial fission. (**A**) Images from live-cell video microscopy demonstrating mitochondrial division in the PfDyn2-3HA^apt^-StrepII-mNeonGreen-Tom22 parasites. The top two rows include images from Video S3 (PfDyn2 sufficient) and the bottom two rows include images from Video S4 (PfDyn2 deficient). Time is displayed as hours:minutes. (**B**) Quantification of PfDyn2-3HA^apt^-StrepII-mNeonGreen-Tom22 parasites that demonstrated mitochondrial fission in PfDyn2-sufficient and -deficient conditions under live-cell video microscopy. Data shown as mean ± SD with individual values overlaid on bar. (**C**) Quantification of individual separated apicoplasts present immediately pre-egress in PfDyn2-sufficient and -deficient PfDyn2-3HA^apt^-StrepII-mNeonGreen-Tom22 parasites during live-cell video microscopy. Data shown as mean ± SD with individual values overlaid on bar. (**D**) Super-resolution microscopy comparing mitochondrial division in PfDyn2-sufficient and -deficient conditions. Late schizont-stage parasites were treated with E64 to block egress prior to fixation. PfMSP1(α-PfMSP1) is in yellow, the apicoplast (α-PfACP) is in green, mitochondria (MitoTracker Orange CMTMRos) are in magenta. Nuclei were stained by Hoechst. (**E**) U-ExM of PfDyn2-3HA^apt^ parasites comparing mitochondrial division in PfDyn2-sufficient and -deficient conditions. Late schizont-stage parasites were pretreated with E64 to prevent egress. Mitochondria (MitoTracker Orange CMTMRos) are in magenta. Maximum projections of approximately 40 z-slices are shown on the left. To the right of each large image are zoomed-in views of selected nascent merozoites (shown as maximum projections of 5–10 z-slices) comparing mitochondrial fission in PfDyn2-sufficient and -deficient schizonts. For the zoomed-in images, the selected merozoites were selected from a subset of z-slices from the larger image. We note that the zoomed-in panels show some overlap of incompletely separated merozoites for the PfDyn2-deficient parasites. Red arrow points to a mitochondrial branch connecting an individualized merozoite to the undivided schizont mass; white arrow points to undivided mitochondrial material adjacent to an enlarged basal complex. All scale bars = 1 µm except for the larger U-ExM images where the scale bar = 5 µm. A–D, data shown are derived from two biological replicates.

Using long-term, live-cell microscopy, we tracked PfDyn2-sufficient and -deficient parasites. PfDyn2-sufficient parasites demonstrated expected patterns of mitochondrial branching and sequential cleavage, with a clear separation of individual mitochondria and merozoites upon egress (Fig. 3A, top rows, Video S3). PfDyn2-deficient parasites consistently failed to divide the mitochondria. Branching structures were formed, but the branched mitochondrial network failed to segment beyond one or two branch cuts (Fig. 3A, bottom rows, Video S4). Extended imaging of the depicted schizont after the initial point of egress (Fig. 3A, bottom rows, 6:00) demonstrates how failure of mitochondrial segmentation hinders separation of individual merozoites, with the primary schizont mass appearing unchanged for over an hour following egress (Fig. 3A, bottom rows, 7:20). We quantified this phenotype by examining >100 parasites that progressed to egress during imaging. PfDyn2-sufficient parasites (93% ± 3.5% per spatial point) showed obvious mitochondrial segregation immediately pre- or post-egress, whereas very few PfDyn2-deficient parasites did (1.6% ± 2.2% per spatial point) (Fig. 3B). Again, we made use of super-resolution, live-cell microscopy to determine when during the process of mitochondrial fission PfDyn2 is required by counting the number of individual, separated, or branched mitochondrial structures present in selected parasites immediately pre-egress. In PfDyn2-sufficient parasites, the mean number of mitochondria visible at this time point was 18 ± 2 (Fig. 3C). PfDyn2-deficient parasites only had 2 ± 1 visibly separated branched mitochondrial structures present immediately pre-egress. As with the apicoplast phenotype, in PfDyn2-deficient parasites, nearly half (48%) of parasites examined failed to separate any branches of the mitochondrial structure and only 13% of the parasites examined had undergone more than one fission event resulting in more than two separated branched mitochondrial structures before egress.

We next used immunofluorescence assays and U-ExM to examine mitochondrial fission at increased resolution in fixed cells. Immunofluorescence utilizing MSP1 again demonstrates that a large, undivided mitochondrial structure is present in the center of the PfDyn2-deficient parasite and that this structure, as well as the majority of the nuclei, is encircled in a single MSP1-enclosed structure rather than individual nuclei being separated by MSP1 as in the PfDyn2-sufficient condition, indicative of a severe failure of segmentation (Fig. 3D). Interestingly, staining with the ACP antibody as well as MitoTracker illustrates that the undivided apicoplast and mitochondrial structures are present in the same region of the schizont and that the few merozoites separated from the mass of nuclei lack both organelles, emphasizing that PfDyn2 is required for the proper segmentation of both structures (Fig. 3D). U-ExM corroborated this observation as PfDyn2-sufficient parasites’ mitochondria separated, such that each merozoite contained a single mitochondrion (Fig. 3E). In PfDyn2-deficient parasites, however, the mitochondrial structure failed to separate, with a mass on one side of the schizont retaining unseparated branches reaching through the basal complexes and retaining connections within the schizont, preventing segmentation (Fig. 3E). As in our immunofluorescence data, some merozoites that lack mitochondria successfully separate from the mass, but the presence of mitochondrial “branches” reaching into multiple merozoites (Fig. 3E, red arrow) illustrates how mitochondrial fission failure, in conjunction with apicoplast fission failure, results in a broad failure of merozoite individualization. The agglomeration of merozoites cannot separate, forcibly connected by branches of undivided mitochondria. We occasionally saw undivided mitochondria adjacent to basal complexes that had failed to fully contract, further demonstrating how mitochondrial division defects cause segmentation failure (Fig. 3E, white arrow). Altogether, these results strongly suggest that PfDyn2 mediates mitochondrial fission and that, as in apicoplast fission processes, PfDyn2 is required for the earliest steps of mitochondrial fission to be completed effectively.

### PfDyn2 is required for effective segmentation

Schizogony in malaria parasites is a highly coordinated, complex process that involves organellar fission as well as the division of one mother cell into 16-32 daughters (33, 35). To evaluate if other defects in cellular

division and segregation beyond the apicoplast and mitochondria were present in parasites with PfDyn2 deficiency, we visualized the apical organelles (via the rhoptry neck marker, PfRON4 [75]), the endoplasmic reticulum (via PfBiP [76]), the Golgi (via PfERD2 [77]), and the centriolar plaques (Fig. S7). We do not observe consistent defects in the segregation or division of these organelles. The morphology of the endoplasmic reticulum was abnormal in a subset of PfDyn2-deficient schizonts (Fig. S7). However, given the defects in segmentation discussed below, it remains unclear if these abnormalities are primary or secondary.

The IMC, a series of flattened vesicles strengthened by a subpellicular network of proteins and located beneath the parasite plasma membrane, acts as a scaffold, providing structural support to each new merozoite. The basal complex, situated at the posterior end of newly formed merozoites, guides IMC formation and is thought to provide the contractile force for cell division (78). Our observations of uncontracted basal complexes adjacent to undivided mitochondria and apicoplasts, and of mitochondrial branches connecting individualized merozoites to the broader schizont mass led us to examine the IMC and basal complex in PfDyn2-deficient parasites.

**Fig 4:**
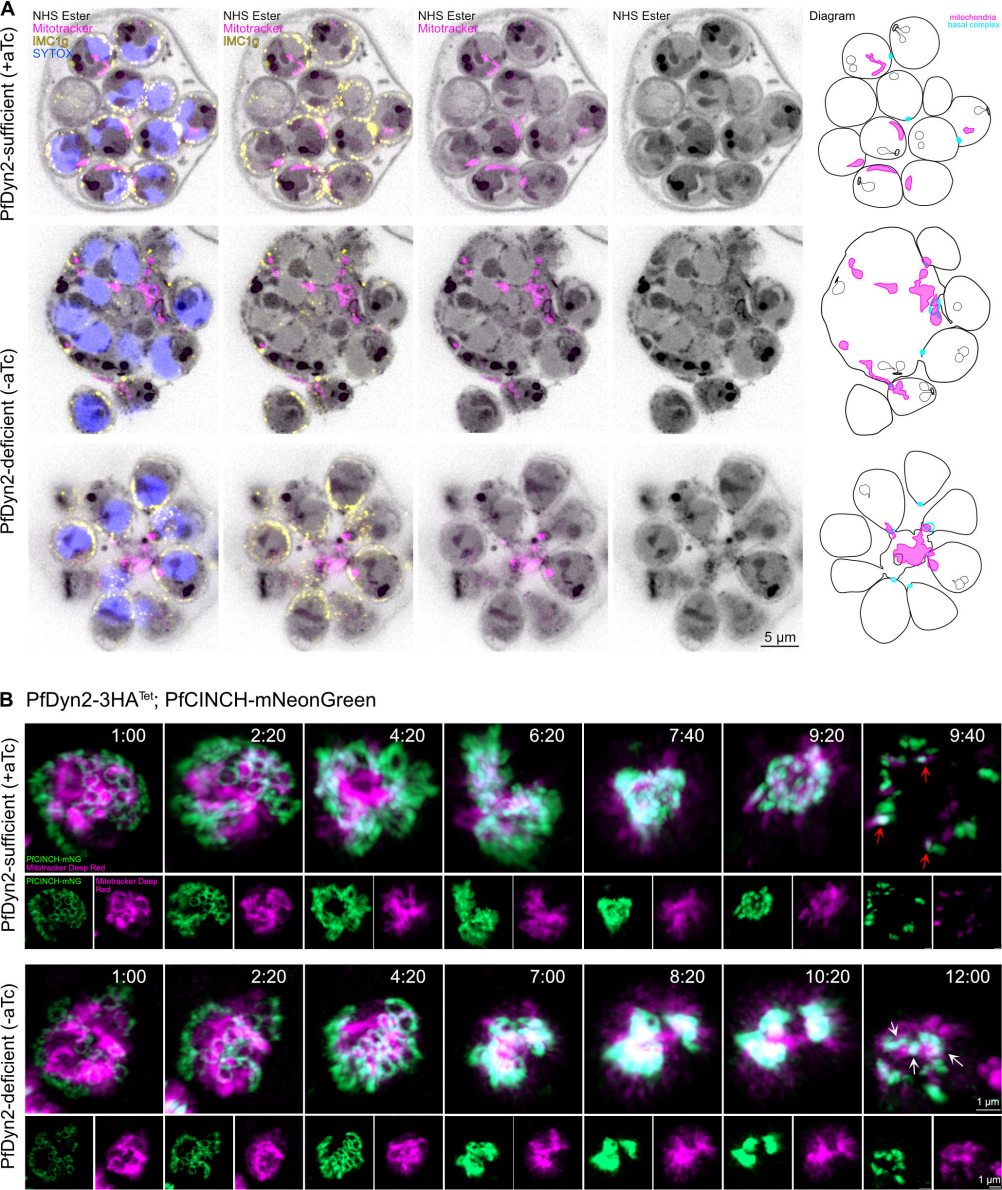
PfDyn2-deficient merozoites fail to complete segmentation. (**A**) U-ExM of PfDyn2-3HA^apt^ parasites comparing segmentation in PfDyn2-sufficient and -deficient conditions. Late schizont-stage parasites were pretreated with E64 to prevent egress. The far-right column contains a diagram to help visualize the size and shape of each visible basal complex in the parasite; in the PfDyn2-deficient condition, amitochondrial merozoites are separated from the residual body but merozoites that contain mitochondria are stuck together. The IMC (α-PfIMC1g) is in yellow, the mitochondria (MitoTracker orange CMTMRos) are in in magenta, and nuclei (SYTOX) are in blue. AF405 NHS Ester was used as a general protein stain and is shown in gray. For clarity of visualization, maximum projections of z-stack images (10–20 slices) are shown. It is important to note that these images contain incomplete merozoites, with portions of the merozoites outside of the selected stacks. Maximum projections of the entire schizonts are shown in the figure. (**B**) Images from live-cell video microscopy in the PfDyn2-3HA^apt^-PfCINCH-mNeonGreen parasites comparing basal complex contraction in PfDyn2-sufficient and -deficient conditions. The top rows include images from Video S5 (PfDyn2 sufficient) and the bottom rows include images from Video S6 (PfDyn2 deficient). The basal complex (PfCINCH-mNG) is in green, the mitochondria (MitoTracker Deep Red) are in magenta. Time is displayed as hours:minutes. In PfDyn2-sufficient conditions, upon egress (time point 9:40), the mitochondria separate along with a contracted PfCINCH-basal complex ring (red arrows). In PfDyn2-deficient conditions, upon egress (time point 12:00), the PfCINCH rings remain stuck on the mitochondria threaded through them, preventing complete contraction (white arrows). Scale bars in (A) = 5 µm; scale bars in (B) = 1 µm. A and B, data shown are derived from two biological replicates.

We first utilized U-ExM and an antibody against an IMC-associated alveolin protein, PfIMC1g (79). PfIMC1g is part of the subpellicular network, which lays directly under the IMC, and it adopts a similar staining pattern to IMC-associated proteins like PfGAP45. In PfDyn2-sufficient conditions, PfIMC1g localizes around individual merozoites, each containing one mitochondrion, one nucleus, and a fully contracted basal complex (Fig. 4A). PfDyn2-deficient parasites, however, displayed severe segmentation defects (Fig. 4A). The middle row of Fig. 4A depicts one example of this defect, where many merozoites cannot be distinguished from each other and larger basal complexes are “clogged” with undivided mitochondria (and presumably undivided apicoplast), prohibiting full contraction of the structure. A few amitochondrial merozoites are visible, separated from the mass and individually surrounded by PfIMC1g, with fully contracted basal complexes. In PfDyn2-deficient parasites, there are differences in basal complex size and shape between merozoites. The bottom row of Fig. 4A shows a slightly different example of this phenotype: each merozoite is surrounded by PfIMC1g, but most mitochondrial material is in the residual body, and basal complexes of merozoites with mitochondria fail to fully contract. However, in the few amitochondrial merozoites, the basal complex can complete the process of contraction, indicating that occlusion of the basal complex by undivided mitochondrial (and likely undivided apicoplast) material prevents full contraction of the basal complex and separation of the associated merozoites from the schizont mass (Fig. 4A; Fig. S8). Although the exact timing of mitochondrial fission relative to the final contraction of the basal complex remains incompletely resolved, U-ExM of PfDyn2-sufficient parasites stained with MitoTracker and antisera against PfIMC1g (Fig. S9) demonstrate that the majority of mitochondrial fission is completed prior to full contraction of the basal complex.

To observe this process occurring in live parasites, we tagged the basal complex protein PfCINCH (80) with mNeonGreen in the PfDyn2-3HA^apt^ background and generated the parasite line PfDyn2-3HA^apt^-PfCINCH-mNeonGreen. We then cultured synchronized parasites with and without aTc and performed long-term, live-cell microscopy after treatment with MitoTracker. In PfDyn2-sufficient parasites, basal complexes synchronously contract around the branched mitochondria as the mitochondria separate (Fig. 4B, top rows; Video S5). In PfDyn2-deficient parasites, basal complex initiation and expansion are not affected, but the basal complex fails to fully contract. Mitochondrial staining remains in the center of the schizont; upon egress, mitochondrial branches do not separate, and PfCINCH rings remain slightly open, threaded onto connected branches of intact mitochondrial material (Fig. 4B, bottom rows; Video S6). Together, these data demonstrate that mitochondrial fission failure in the absence of PfDyn2 causes broader segmentation failure by preventing the full contraction of the basal complex, which remains occluded by mitochondrial material, which in turn prevents individualization and separation of merozoites. It is important to note that the similar defect in apicoplast fission (Fig. 2) likely contributes to the segmentation defect as well.

## DISCUSSION

In this work, we characterize PfDyn2, a novel dynamin-related protein. PfDyn2 localizes to the apicoplast and mitochondria, starting in mid-schizogony, and is essential for asexual replication. Importantly, PfDyn2-deficient parasites failed to be rescued by IPP, indicating that PfDyn2 is required for multiple processes, rather than just ensuring apicoplast function in new merozoites. Using super-resolution, long-term, live-cell, and ultrastructure expansion microscopy, we demonstrate that PfDyn2 is required for apicoplast and mitochondrial fission, as PfDyn2-deficient parasites cannot separate these organelles. Indeed, our live-cell imaging data allow us to determine that PfDyn2 is required for the earliest stages of mitochondrial and apicoplast fission, as its depletion results in most parasites egressing with only one or two distinct organellar structures. Thus, even the initial cuts separating branches of the mitochondria and apicoplast from each other cannot be carried out efficiently in PfDyn2-deficient parasites.

We have demonstrated that preventing organellar fission in *P. falciparum* has severe consequences for segmentation. In PfDyn2-deficient conditions, the basal complex fails to fully contract because undivided mitochondria and apicoplasts occlude the ring itself, a conclusion bolstered by the fact that, in some merozoites that lack mitochondria, the basal complex is fully contracted at the end of segmentation (Fig. 4). IMC distribution is impacted by this as well: failure of mitochondrial and apicoplast division leads to the IMC encircling an undifferentiated mass of cellular material. In these parasites, only a few amitochondrial merozoites are individually packaged. Thus, because the branched mitochondrial and apicoplast structures during schizogony are centered in the residual body, failure of organellar fission prevents segmentation even though basal complex integrity is not compromised. In contrast, defects of basal complex contraction do not seem to impact organellar fission—parasites deficient in PfCINCH, required for basal complex contraction, still possessed segmented apicoplast and mitochondria, despite the accumulation of cell materials in large “megazoites” (80). The IMC and basal complex thus do not actively divide the organelles in *Plasmodium*, and their defects do not prevent organellar fission. Furthermore, our results suggest that the rhoptries, endoplasmic reticulum, Golgi apparatus, and centriolar plaques appear to divide normally in PfDyn2-deficient parasites. Nonetheless, it remains possible that PfDyn2 may be contributing to more general organellar division defects beyond the mitochondria and apicoplast that were not evident in our analysis.

Our data clearly show that PfDyn2 has distinct roles that are not present in its closest ortholog in *T. gondii,* TgDrpA. Of the three dynamin-related proteins in *P. falciparum*, PfDyn1 is thought to play a role in hemoglobin uptake (53, 81), PfDyn2 is required for apicoplast and mitochondrial division (this study), and PfDyn3 remains unknown. Immunoprecipitation of basal complex proteins (82) detected the presence of PfDyn2, indicating PfDyn2 could be involved in basal complex contraction or performing the final scission of merozoites in lieu of ESCRT-III machinery (83–85), which apicomplexan parasites lack. However, in PfDyn2-deficient parasites, we observed that the few amitochondrial merozoites that separate from the schizont have fully contracted basal complexes (Fig. 4A), suggesting that PfDyn2 is not directly involved in basal complex contraction. PfDyn2 is likely present in basal complex co-immunoprecipitation data (80, 82) because organellar fission occurs as the basal complex contracts and PfDyn2, present on the outer membrane of both the apicoplast and the mitochondrion, is physically near the basal complex. Although the three dynamin orthologs are present in *T. gondii*, a related apicomplexan parasite, their roles appear to be different. TgDrpA, the homolog of PfDyn2, is required for apicoplast division, but its depletion does not significantly impact mitochondrial fission (49), whereas PfDyn2 acts on both organelles in *Plasmodium*. TgDrpB is involved in secretory organelle biogenesis (50), and TgDrpC is required for mitochondrial division (52), with TgDrpC-deficient parasites exhibiting interconnected mitochondria like PfDyn2-deficient schizonts. TgDrpC was also suggested to play broader roles in cytokinesis and organellar biogenesis beyond mitochondrial fission (51). Our work demonstrates that even orthologous dynamin-related proteins have distinct biological functions in different apicomplexan parasites such as *Plasmodium* and *Toxoplasma*. This could be, at least partially, a functional differences in organellar structure between these two parasites. In *Plasmodium*, the mitochondrion and apicoplast are both small, compact globular structures during the ring and trophozoite stages (Fig. 2 and 3; Fig. S5 and S6) (30). These organelles only gain more structural complexity as they begin to expand and divide during schizogony, and it has been shown that the mitochondrion and apicoplast physically associate in the asexual blood stages, retaining points of contact throughout asexual development (30). In contrast, the *Toxoplasma* mitochondrion forms a large, lasso-like structure around the periphery of the parasite during interphase, whereas the apicoplast remains small and does not form a large, branched structure at any point during the parasite’s replicative cycle (86, 87). Nonetheless, interactions between the apicoplast and mitochondrion has also been observed in *T. gondii* in multiple studies, and these interactions suggest a biochemical and metabolic relationship between the two organelles in this organism (88). Altogether, unlike TgDrpA that is only responsible for apicoplast fission, PfDyn2 executes distinct roles in *P. falciparum* by mediating both mitochondrial and apicoplast fission.

Since *P. falciparum* asexual division via schizogony differs from *T. gondii* tachyzoite division via endopolygeny in many important ways, we notice interesting differences between the biology of *Toxoplasma* and that of *Plasmodium*. TgDrpA is required for apicoplast fission, and it was hypothesized that the force generated by daughter bud growth—i.e., by the expansion and contraction of the basal complex—constricted the apicoplast initially while TgDrpA performed the final separation (49, 89). Parasites defective in TgMORN1, an essential component of *Toxoplasma*’s basal complex (90), fail to both constrict the basal complex and divide the apicoplast (89). Interestingly, the TgMORN1 knockout had a milder segregation defect for the mitochondrion compared to the apicoplast, suggesting differential involvement of the basal complex in division of each organelle. In *T. gondii*, the mitochondrion interacts with the pellicle throughout its life cycle, and the apicoplast contacts the pellicle specifically during endodyogeny (91, 92). It makes sense then that basal complex contraction is required for apicoplast division and the final stage of mitochondrial fission in *Toxoplasma* (93). The fact that *Plasmodium* mitochondria and apicoplast seem to lack contacts with the pellicle and basal complex during much of their growth and division (because these structures are absent in rings and trophozoites) may explain why mitochondrial and apicoplast fission defects impact segmentation in *Plasmodium*, but not the other way around (35, 94). Because these endosymbiotically derived organelles have not definitively been shown to form contacts with the pellicle or basal complex in *Plasmodium*, their division may not rely on these structures but utilizes a different set of proteins. Hence, we observe different apicoplast and mitochondrial phenotypes in PfCINCH-deficient schizonts (80) compared to TgMORN1-deficient tachyzoites (89).

Despite having identified the dynamin-related proteins providing the force for organellar fission in both *Plasmodium* and *Toxoplasma*, no homologs to known receptors or recruitment proteins (like Mff or Mid49/51) have been identified in either organism (2). PfFis1, a *Plasmodium* homolog of a mitochondrial outer membrane adapter protein, is dispensable (37), as is the *T. gondii* homolog TgFis1 (95), although both localize to the mitochondria. One possibility for the dispensability of PfFis1/TgFis1 is that apicomplexan parasites have evolved divergent mitochondrial outer membrane and/or apicoplast-binding adaptor proteins. In *Plasmodium*, these adaptor proteins could be common to the apicoplast and mitochondria because PfDyn2 acts on both, making them less likely to be direct homologs of known mitochondrial outer membrane adaptor proteins of model organisms. Further studies, like applying a proximal-biotinylation approach to PfDyn2, could identify putative adaptor proteins common to both endosymbiotically derived organelles and adaptor proteins unique to each one. In addition to PfDyn2 and receptor proteins yet to be found, a previous study revealed the role of actin in apicoplast fission/segregation and cytokinesis (96). Interestingly, actin depletion only impairs apicoplast fission but not mitochondrial fission, indicating that although PfDyn2 is required for both processes, there are additional, distinct molecular players needed to mediate apicoplast fission and mitochondrial fission in *P. falciparum*. It remains formally possible that the fitness defects observed for PfDyn2-deficient parasites are due to loss of secondary interactions with other critical proteins. Moreover, we have not directly evaluated the mode of action of PfDyn2 in this work. When expressed recombinantly, PfDyn2 is known to have GTPase enzymatic activity, as predicted bioinformatically (55). Although we hypothesize that this enzymatic activity is required for the function of PfDyn2 in replicating *P. falciparum,* genetic study with attempted complementation by a catalytically inactive version of the protein is required to definitively test this hypothesis. In addition, although it has been established that during late segmentation, the apicoplast divides approximately 1–2 hours ahead of the mitochondrion, we could not confidently determine whether PfDyn2 localizes to the apicoplast before the mitochondrion (30). Utilization of higher-resolution microscopy techniques of live cells or implementation of techniques that mechanically increase functional resolution, like iterative- or Pan-ExM (97), may potentially demonstrate an order of recruitment for PfDyn2. However, we currently cannot rule out the possibility that PfDyn2 binds the apicoplast and mitochondria simultaneously with other factors enabling division of the apicoplast before the mitochondrion.

In summary, PfDyn2 localizes to and is essential for the division of both the apicoplast and the mitochondrion. For the first time, we utilize super-resolution, long-term, live-cell imaging to visualize apicoplast and mitochondrial division over the entirety of schizogony, allowing us to conclude that PfDyn2 specifically acts during the earliest stages of organellar division, as its removal prohibits even the initial separation of organellar branches. Our ability to visualize the basal complex and mitochondrion simultaneously during segmentation allowed us to determine precisely how organellar fission failure leads to broader segmentation defects: the basal complexes of newly forming merozoites remain connected by undivided organellar material, preventing full contraction of the complex and individualization of merozoites. Although additional studies are needed to identify adaptor proteins and characterize pathways of mitochondrial and apicoplast division in *P. falciparum*, we have already uncovered data that suggest endosymbiotic organellar division in *Plasmodium* occurs via a significantly different mechanism than in other eukaryotes, including the closely related apicomplexan parasite, *T. gondii*.

## MATERIALS AND METHODS

### Plasmid construction

#### Conditional knockdown of PfDyn2 using the TetR-DOZI-aptamer system

The two homologous regions of PfDyn2 (PF3D7_1037500, 3HR, 5HR) were PCR amplified from genomic DNA using primers P1/P2 (3HR) and P3/P4 (5HR). The DNA fragments were sequentially cloned into the pMG75-BSD-3HA construct (98) and verified by Sanger sequencing using vector primers (P5 and P6). These procedures produced pMG75-BSD-PfDyn2-3HA, which was linearized with EcoRV prior to transfection. Two gRNA coding sequences (P7, P8) were selected from near the end of the PfDyn2 genetic locus by Eukaryotic Pathogen CRISPR guide RNA/DNA Design Tool (grna.ctegd.uga.edu). They were individually cloned into the NFCas9 plasmid with infusion as described previously (99), yielding two gRNA expressing plasmids. The gRNA sequences were verified by Sanger sequencing (P9).

#### Labeling of the mitochondria with mNeonGreen (mNG)

We fused mNG to the N-terminus of Tom22 (PF3D7_0524700). We also added a twin Strep tag upstream of mNG-Tom22, which can facilitate mitochondrial purification. Starting from pLN-Cam-BSD-HSP60L-mNG, we used Infusion to replace the HSP60L sequence with a synthetic gene block containing the Strep II sequence (P18), yielding pLN-Cam-BSD-StrepII-mNG. Colony PCR was performed to select positive clones (P19/P17). The correct sequence of StrepII-mNG was verified by sequencing (P20). Next, pLN-Cam-BSD-StrepII-mNG was further digested with BsrGI and AflII and ligated with Tom22, which was amplified from genomic DNA using primers P21/P22. The correct Tom22 sequence was verified by Sanger sequencing (P17). These procedures yielded pLN-Cam-BSD-StrepII-mNG-Tom22. To switch the selectable marker from BSD to hDHFR, StrepII-mNG-Tom22 was excised by AvrII and AflII and subsequently cloned into two pLN plasmids, pLN-RL2-hDHFR-RL13-3Myc (100) and pLN-Cam-hDHFR-VP1-3Myc (98). These procedures produced two plasmids, pLN-RL2-hDHFR-StrepII-mNG-Tom22 and pLN-Cam-hDHFR-StrepII-mNG-Tom22. Transfections of both plasmids were conducted; however, only parasites transfected with pLN-RL2-hDHFR-StrepII-mNG-Tom22 had viable parasites and proper labeling of mitochondria.

#### Labeling of the apicoplast with mRuby

We PCR amplified mRuby from PM2GT-mRuby-Hsp101 using primers P23/P24 and subsequently cloned it into pLN-RL2-hDHFR-RL13-3Myc (100) using AvrII and AflII, resulting in pLN-RL2-hDHFR-mRuby. The leader sequence of ACP (first 55 aa) was PCR amplified from genomic DNA using primers P25/P26 and cloned into pLN-RL2-hDHFR-mRuby by AvrII and NheI, yielding pLN-RL2-hDHFR-ACP_L_-mRuby. The correct sequence of ACP_L_-mRuby was verified by Sanger sequencing (P16).

### Parasite culture and transfection

Wild-type *P. falciparum* strains, D10 and NF54attB, were used in the study. Parasites were grown in complete RPMI-1640 media supplemented with Albumax I (0.5%) and human O^+^ RBCs, as previously described (100). Anonymous human RBCs were purchased from commercial vendors (Interstate Blood Bank or BioIVT). Our institutional review boards have determined that this is not considered human subjects research. Transfections were performed in ring-stage parasites (>5% parasitemia) with either linearized or circular plasmids (~50 µg) using a Bio-Rad electroporator. Post-electroporation, the cultures were kept in a low oxygen atmosphere (90% N_2_, 5% CO_2_, 5% O_2_) and added with proper drug selections, e.g., blasticidin (2.5 µg/mL, InvivoGen), WR99210 (5 nM, Jacobs Pharmaceutical), G418 (125 µg/mL, VWR), or aTc (250 nM, Fisher Scientific).

To construct the line PfDyn2-3HA^apt^-PfCINCH-mNeonGreen, 25 µg of PfCINCH-HDR plasmid containing mNeonGreen (pRR208) was linearized by digestion with STUI, purified, and co-transfected with 20 µg of a guide plasmid (pRR99), a construct containing an SpCas9 expression cassette and the PfCINCH-targeting guide RNA. Transfection was performed in the NF54attB-Dyn2-3HA^apt^ line. Parasites were maintained with 2.5 µg/mL blasticidin and 500 nM aTc from the onset of transfection. One day post-transfection, drug pressure for the second marker was applied with 5 nM WR99210.

### Knockdown studies/Western blot analysis

To initiate knockdown studies, the synchronized NF54attB-PfDyn2-3HA^apt^ parasites at the ring stage were thoroughly washed with 1× phosphate-buffered saline (PBS) to remove aTc and were diluted in fresh RBCs to receive aTc (+) or (–) media. For regular knockdown studies, thin blood smears and parasite proteins were harvested daily or every 2 days. For time course knockdown studies, samples were collected every 4 hours for six times, starting at 24-hour post-aTc removal. To extract proteins, the parasite cultures were treated with 0.05% Saponin/PBS supplemented with 1× protease inhibitor cocktail (Apexbio Technology LLC), and the pellets were solubilized by 2% SDS/Tris-HCl (65 mM, pH 6.8). The other Western blot procedures followed standard protocols. The blots were incubated with primary antibodies, including the HA probe (mouse, sc-7392, Santa Cruz Biotechnology; 1:10,000) and anti-PfExp2 (rabbit, a kind gift from Dr. James Burns, Drexel University; 1:10,000). Secondary horeseradish peroxidase-labeled antibodies were purchased from Thermo Fisher Scientific, including goat anti-mouse (A16078, Thermo Fisher Scientific, 1:10,000) and goat anti-rabbit antibody (31460, Thermo Fisher Scientific, 1:10,000). The blots were incubated with Pierce ECL substrates and were developed by the ChemiDoc Imaging Systems (Bio-Rad).

### Immunofluorescence analysis (IFA) with Nikon Ti

NF54attB-PfDyn2-3HA^apt^ parasites were tightly synchronized with several rounds of alanine/HEPES (0.5 M/10 mM). In various stages (ring, trophozoite, schizont), aliquots of parasite cultures were removed (~50 µL per aliquot). Prior to being fixed with 4% formaldehyde/0.0075% glutaraldehyde, they were either labeled with 60 nM of MitoTracker Red CMXRos (M7512, Thermo Fisher) for 30 minutes or left untreated. After fixation, the cells were permeabilized with 0.25% Triton X-100/PBS, reduced with NaBH4 (0.1 mg/mL), blocked with 3% bovine serum albumin (BSA)/PBS. The cells were incubated with primary antibodies, such as the HA probe (mouse, sc-7392, Santa Cruz Biotechnology; 1:300) and/or anti-PfACP (rabbit, a kind gift from Dr. Sean Prigge, 1:500). Fluorescently labeled secondary antibodies were purchased from Life Technologies (Thermo Fisher Scientific) (anti-mouse or anti-rabbit, 1:300). The samples were stained with 4’,6-diamidino-2-phenylindole (DAPI) for 10 minutes (1.5 µg/mL in PBS) and mounted on glass slides in antifade buffer (S2828, Thermo Fisher Scientific). Images were captured using a Nikon Ti microscope and were processed using the Nikon NIS elements software.

### Immunofluorescence analysis (IFA) with Zeiss LSM900 AiryScan2

Schizonts were percoll purified, resuspended in 100 µL media (with or without 300 nM MitoTracker Orange CMTMRos [M7510, Thermo Fisher]), and placed on poly-D-lysine-treated 10-mm diameter #1.5 coverslips to settle for 25 minutes at 37°C in one well of a 24-well plate. Excess/unbound cells were removed, and 300 µL of 4% paraformaldehyde (PFA) in PBS was added for 20 minutes of fixation. PFA was removed, and the coverslip was washed three times with 1× PBS. Three hundred microliters of 0.1% Triton-X 100 in PBS was then added to the well for 10 minutes to permeabilize the cells. Coverslip was washed three times for 3 minutes with 1× PBS following permeabilization. BSA (3%) in PBS was added for blocking for 1 hour, after which relevant primary antibodies resuspended in 3% BSA in PBS were added, and the coverslips were incubated overnight at 4°C. Coverslips were then washed three times with 1× PBS for 3 minutes again, and secondary antibodies were added for 45 minutes in the dark at room temperature (RT), diluted 1:1,000 in 0.5% BSA in PBS. Coverslips were again washed three times for 3 minutes with 1× PBS. For 10 minutes, a 1:5,000 dilution of Hoechst 3342 in 1× PBS was added to the coverslips. Coverslips were then rinsed with 1× PBS one more time before they were adhered to slides with 5 µL of VectaShield Vibrance (hardening, non-DAPI). IFAs were visualized at least 3–4 hours after mounting. Cells were visualized on a Zeiss LSM900 AiryScan 2 for super-resolution microscopy, with a 63× magnification objective with a numerical aperture of 1.4.

Rat anti-HA (used at 1:500) was purchased from Sigma (catalog 11867423001), mouse anti-CrCen (used at 1:250) was purchased from EMD Millipore (catalog 04-1624), rabbit anti-PfGAP45 (used at 1:5,000) was a generous gift from Julian Rayner at the Cambridge Institute for Medical Research, rabbit anti-PyACP (used at 1:1,000) was a generous gift from Scott Lindner at Pennsylvania State University, rabbit anti-PfERD2 was obtained from BEI Resources (MRA-1, used at 1:200), and mouse anti-PfRON4 was a generous gift from Alan Cowman at Walter and Eliza Hall Institute (used at 1:200). Mouse anti-MSP1 (used at 1:500) was a generous gift from Anthony Holder at the Francis Crick Institute. Rabbit anti-PfBiP (used at 1:500) and rabbit anti-PfIMC1g (used at 1:1,250) were generated previously (79, 94). All secondary antibodies (anti-rat AF488, anti-rat AF647, anti-rabbit AF488) were purchased from Life Technologies and used at 1:1,000.

### Ultrastructure expansion microscopy

Synchronized Dyn2-3HA^apt^ aTc (±) schizonts were percoll purified as for standard immunofluorescence. Isolated schizonts were placed on poly-D-lysine-coated coverslips for 20–30 minutes at 37°C to settle in one well of a 24-well plate (with 300 nM MitoTracker Orange CMTMRos). Parasites were then fixed with 4% PFA for 20 minutes at 37°C and were washed three times with PBS. Following fixation, the coverslips were incubated with formaldehyde/acrylamide (FA/AA) overnight at 37°C. The next morning, to polymerize the gel, N,N,N’,N’-tetramethylethylenediamine (TEMED) and ammonium persulfate (APS) were swiftly added to a previously made monomer solution containing acrylate, acrylamide, and N,N’-methylenebisacrylamide (BIS). The monomer solution was placed as a droplet inside a humid chamber stored at −20°C for 15 minutes preceding gelation. Coverslips were then placed over this drop of the TEMED/APS/monomer solution. After 5 minutes of incubation on ice, the chamber was incubated at 37°C for an hour. Coverslips/gels were then placed in 2 mL of denaturation buffer (in one well of a 6-well plate) and were incubated with denaturation buffer for 15 minutes with agitation at room temperature. After gel detachment, gels were incubated for 90 more minutes at 95°C in a 1.5-mL Eppendorf tube filled with denaturation buffer. Denaturation buffer was then removed, and gels were placed in a 10-cm dish filled with ddH2O. ddH2O was replaced after 30 minutes, twice. Gels were then incubated in ddH2O overnight. The next morning, gels were washed in 1× PBS two times for 15 minutes each then incubated in 3% BSA-PBS for 30 minutes at RT. Blocking buffer was removed, and gels were incubated in 1 mL of 3% BSA-PBS with primary antibodies overnight at 4°C. Primary antibodies were used at the following dilutions: rat anti-HA (1:250), rabbit anti-PyACP (1:500), rabbit anti-PfIMC1g (1:1,000). The next day, gels were washed three times with 2 mL 0.5% Tween20 in PBS for 10 minutes at room temperature with agitation. Gels were then incubated in 1 mL of PBS with secondary antibodies including NHS-Ester (AlexaFluor 405, 1:100) protected from light, with agitation at room temperature. After 2.5 hours of secondary antibody incubation, gels were washed three more times with 2 mL 1% PBS + 0.5% Tween20 as before, then placed in a 10-cm dish filled with ddH2O. Water was replaced after 30 minutes, twice, and gels were allowed to expand overnight before imaging on a Zeiss LSM900 AiryScan 2 for super-resolution microscopy, with a 63× magnification objective and a numerical aperture of 1.4.

### Long-term, live-cell imaging

Parasites were synchronized by means of sorbitol treatment and percoll density centrifugation one or two cycles (2 or 4 days) prior to the experiment. On the day of the experiment, schizonts were percoll purified and placed in a 5-mL (mm) culture dish with 5 mL of complete media to recover for 30 minutes. They were then gently spun down and resuspended in 100 µL of media and placed in one quadrant of a concanavalin A-coated iBidi/cellview glass bottom dish. After 30 minutes of incubation at 37°C, excess cells were washed off with 3× washes of 1× PBS, and each quadrant in use was filled with phenol red-free RPMI with 0.5 mM Trolox added. Parasites were imaged in a 37°C incubation chamber supplemented with 5% CO_2_; the glass bottom dish was also inset into a heated stage (heated to 37°C) on a Zeiss LSM900 AiryScan 2. The SR-Multiplex Mode (4Y) was used, and images were taken every 20 minutes over 13 hours. For quantification, resulting files were visualized in FIJI. To produce supplementary videos and high-resolution images, resulting files were processed in ARIVIS 4D.

For imaging the PfDyn2-3HA^apt^-PfCINCH-mNeonGreen line, MitoTracker Deep Red FM was added to the media to a final concentration of 10 nM during the settling stage to label mitochondria.

Laser use was dependent on the fluorophore or dye utilized for each experiment. For PfDyn2-3HA^apt^-PfCINCH-mNeonGreen imaging (Fig. 4), the 488 laser was used at 0.9% laser power and 850 gain, and the 647 laser was used at 1.0% laser power with 750 gain. For PfDyn2-3HA^apt^-StrepII-mNeonGreen-Tom22 imaging (Fig. 3), the 488 laser was used at 0.9% laser power and 850 gain, and the 647 laser was used for transmitted light/brightfield imaging through ESID. For PfDyn2-3HA^apt^-ACP_L_-mRuby imaging (Fig. 2), the 561 laser was used at 0.9% laser power and 800 gain, and the 647 laser was used for transmitted light/brightfield imaging through the electronically switchable illumination and detection (ESID) module.

For a single time point live-cell imaging of PfDyn2-3HA^apt^-StrepII-mNeonGreen-Tom22 (Fig. S6), MitoTracker Deep Red FM (10 nM) was added for 20 minutes, and Hoechst (1:10,000 dilution of 10 mg/mL) was added for 10 minutes prior to imaging.

### Image quantification

For Fig. 2B, 10 fields per condition were taken with 9–15 parasites per field. Parasites were considered “positive” for successful apicoplast division if, at the time point immediately pre-egress or, that time point was unclear, the time point of immediately after egress, there were visible and obviously separated apicoplasts (see Fig. 2A for an example of this). Only parasites that egressed during the imaging time course were considered part of the data set.

For Fig. 2C, 30–40 parasites (depending on the condition) per condition were selected for quantification of apicoplasts produced. Selection criteria were as follows: stability of parasite in the Z-dimension (drifting up or down makes the number of visible organelles unclear), stability of fluorescence (only parasites that did not bleach significantly between initiation of imaging and egress were considered), and orientation of parasite (if the red blood cell was adhered on its side, for example, it was difficult to tell whether adjacent apicoplasts were still connected or not).

This sample size was more than enough for us to be confident in our quantification: a pilot experiment determined that with the severity of the difference in mean number of apicoplasts between conditions, examining fewer than 10 parasites per condition with an assumed type one error rate of 0.05 would provide a statistical power above 0.99.

After selection of parasites, the time point immediately before egress was identified, and the number of individual apicoplast structures was counted (defined by separation of the structures by multiple black pixels). Both maximum projection and stacked Z-slice images were visualized to ensure accuracy of quantification. If it was unclear whether adjacent apicoplasts were separated, they were counted as one structure.

For Fig. 3B and C (quantification of mitochondria segmentation), five fields per condition were taken with 15–30 parasites per field. Analysis and quantification were performed as for Fig. 2.

## Data Availability

All data are present in the manuscript and supplementary information. Plasmids and parasite strains are available upon request.

## SUPPLEMENTAL MATERIAL

Supplemental Information mbio.03036-24-s0001.pdf Supplemental figures, table, and legends for supplemental videos.

Video S1 mbio.03036-24-s0002.mp4 Three-dimensional rendering of time-lapse confocal (AiryScan Multiplex 4Y) imaging of PfDyn2-3HAapt-ACPL-mRuby (PfDyn2-sufficient condition).

Video S2 mbio.03036-24-s0003.mp4 Three-dimensional rendering of time-lapse confocal (AiryScan Multiplex 4Y) imaging of PfDyn2-3HAapt-ACPL-mRuby parasites (PfDyn2-deficient condition).

Video S3 mbio.03036-24-s0004.mp4 Three-dimensional rendering of time-lapse confocal (AiryScan Multiplex 4Y) imaging of PfDyn2-3HAapt-StrepII-mNeonGreen-Tom22 parasites (PfDyn2-sufficient condition).

Video S4 mbio.03036-24-s0005.mp4 Three-dimensional rendering of time-lapse confocal (AiryScan Multiplex 4Y) imaging of PfDyn2-3HAapt-StrepII-mNeonGreen-Tom22 parasites (PfDyn2-deficient condition).

Video S5 mbio.03036-24-s0006.mp4 Three-dimensional rendering of time-lapse confocal (AiryScan Multiplex 4Y) imaging of PfDyn2-3HAapt-PfCINCH-mNeonGreen parasites (PfDyn2-sufficient condition) stained with 10 nM MitoTracker Deep Red FM.

Video S6 mbio.03036-24-s0007.mp4 Three-dimensional rendering of time-lapse confocal (AiryScan Multiplex 4Y) imaging of PfDyn2-3HAapt-PfCINCH-mNeonGreen parasites (PfDyn2-deficient condition) stained with 10 nM MitoTracker Deep Red FM.

ASM does not own the copyrights to Supplemental Material that may be linked to, or accessed through, an article. The authors have granted ASM a non-exclusive, world-wide license to publish the Supplemental Material files. Please contact the corresponding author directly for reuse.
