# Supplementary material for: The dynamin-related protein PfDyn2 is essential for both apicoplast and mitochondrial fission in Plasmodium falciparum
Source: mBio. 2024 Nov 29;16(1):e03036-24. doi: 10.1128/mbio.03036-24 (PMC11708027; doi:10.1128/mbio.03036-24)
Supplement: Supplemental Information — Supplemental figures, table, and legends for supplemental videos. [file mbio.03036-24-s0001.pdf]

**Supplementary Information**

The dynamin-related protein PfDyn2 is essential for both apicoplast and mitochondrial fission in *Plasmodium falciparum*

Alexander A. Morano[1,2#], Wei Xu[3,#], Francesca M. Navarro[1,2], Neeta Shadija[3], Jeffrey D. Dvorin[1,4,*], Hangjun Ke[3,*]

[#]These authors contributed equally and are ordered alphabetically.

[1]Division of Infectious Diseases, Boston Children's Hospital, Boston, Massachusetts, USA, 02115

[2]Biological and Biomedical Sciences, Harvard Medical School, Boston, Massachusetts, USA, 02115

[3]Center for Molecular Parasitology, Department of Microbiology and Immunology, Drexel University College of Medicine, Philadelphia, Pennsylvania, USA, 19129

[4]Department of Pediatrics, Harvard Medical School, Boston, Massachusetts, USA, 02115

*To whom correspondence should be addressed:

Dr. Jeffrey D. Dvorin – Jeffrey.Dvorin@childrens.harvard.edu

Dr. Hangjun Ke – hk84@drexel.edu

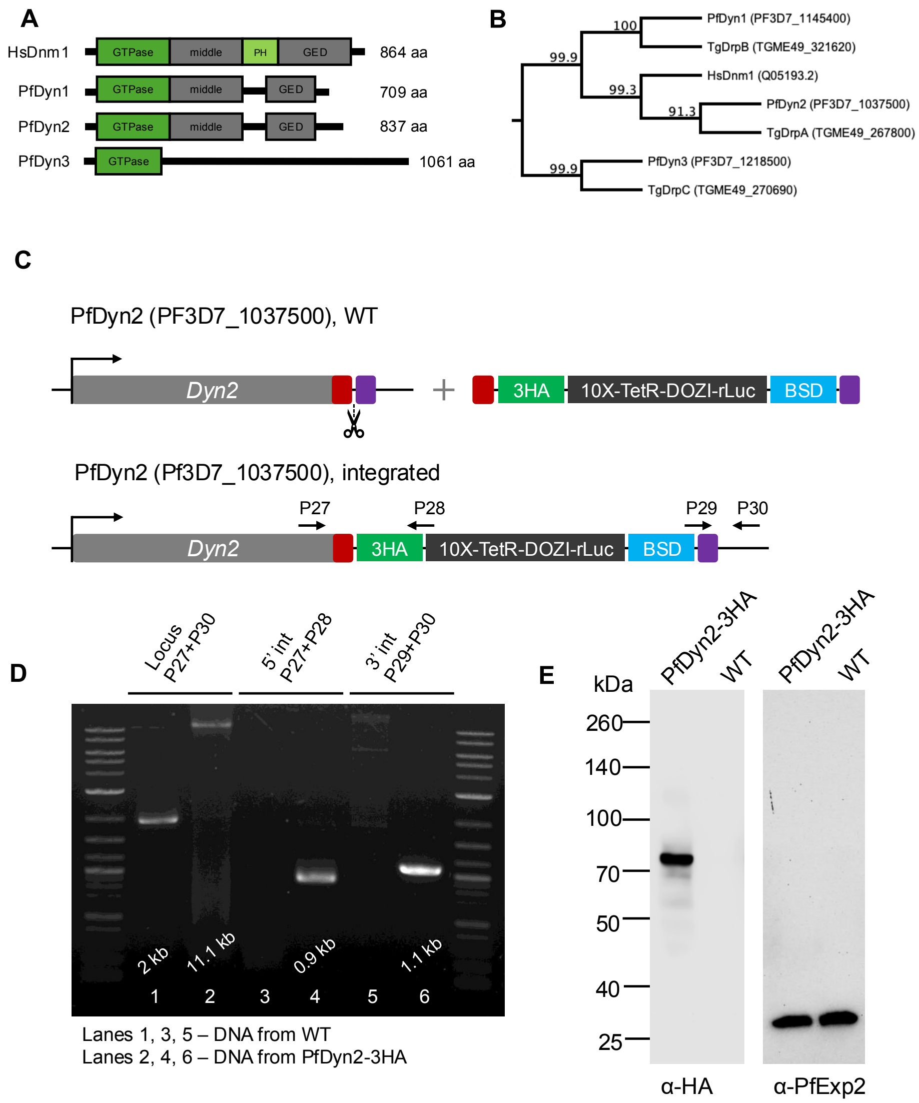


**Figure S1**. Endogenous tagging PfDyn2 via CRISPR/Cas9.

**Figure S1. A**) Major domains of the three dynamin-like proteins in *P. falciparum* (PfDyn1-3). PfDyn1-3 are divergent from human dynamin 1, and they lack a recognizable Pleckstrin homology domain (PH). The location of the GTPase Effector Domain (GED) are shown in each. **B**) Phylogenetic tree of PfDyn1, PfDyn2, PfDyn3, TgDrpA, TgDrpB, TgDrpC, and HsDnm1. Protein sequences were aligned ("Geneious alignment"), and phylogenetic tree was generated using default settings ("Majority greedy clustering" with Geneious Prime v2024.0.7, numbers show consensus support percentages). **C**) Schematic of genetic modification of the *PfDyn2* (PF3D7_1037500) locus. The gene was modified to include a 3HA tag and elements of the TetR-DOZI-aptamer knockdown system. Primers used for diagnostic PCR in **D** are indicated. **D**) PCR genotype shows expected modification of *PfDyn2* locus. Expected sizes for amplicons are as follows: "locus" ~2 kb for parental, ~11.1 kb for transgenic; "5' int" no band for parental, 0.9 kb for transgenic; and, "3' int" no band for parental, ~1.1 kb for transgenic. **E**) Western blot showing the expression of PfDyn2-3HA (expected sizes for PfDyn2-3HA and PfExp2 are 85 kDa and 33 kDa, respectively). WT (wild type) lysate was derived from the parental parasite line, NF54attB. PfExp2 was used as a loading control.

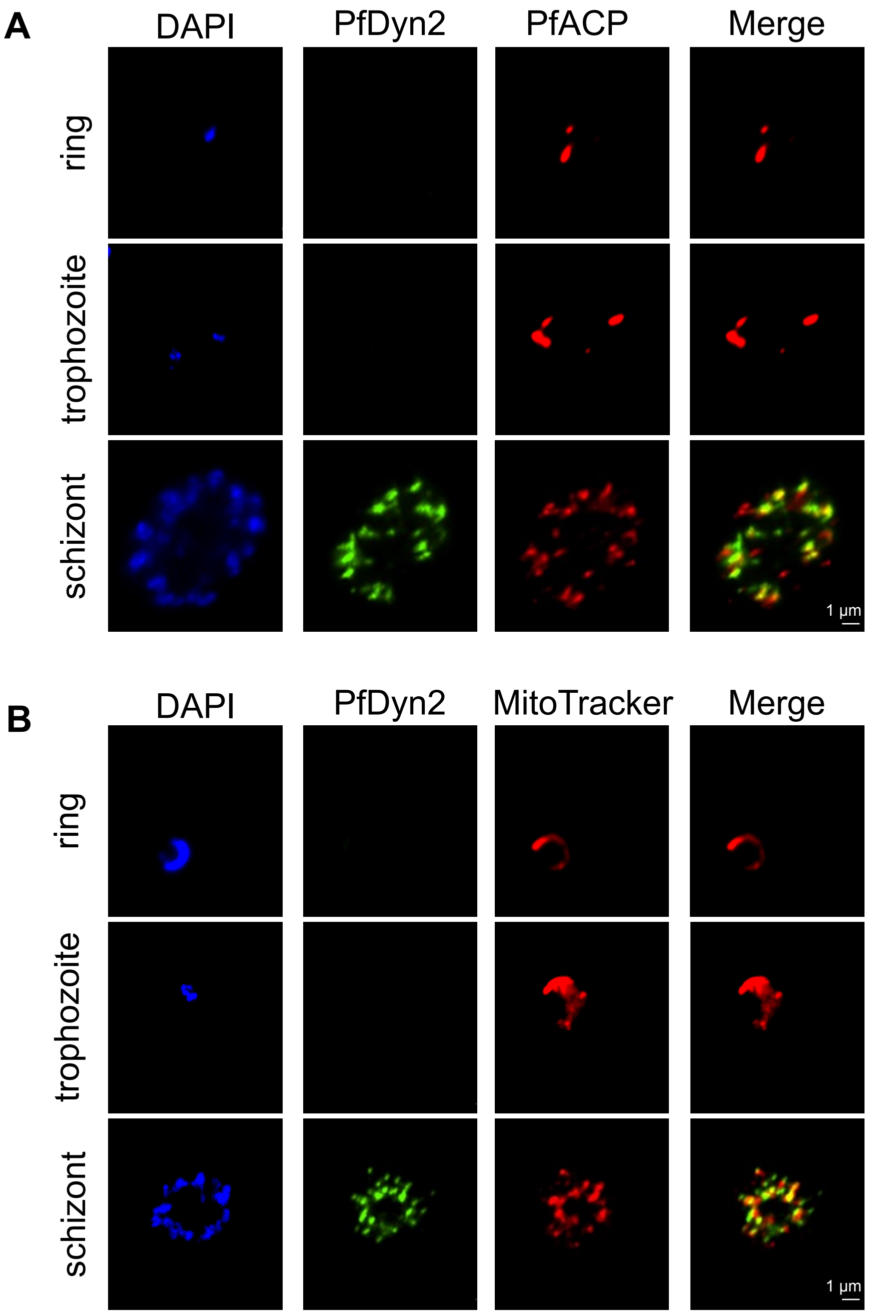


**Supplementary Figure 2**. Localization of PfDyn2 in asexual blood stages

**Figure S2. A**) Immunofluorescence assays (IFA) showing localization of PfDyn2 to the apicoplast in the schizont stage, but not in the ring or trophozoite stages. DAPI stains nucleus. PfDyn2 was detected by α-HA. The apicoplast was labeled with α-PfACP, acyl carrier protein. **B**) Immunofluorescence assays (IFA) showing localization of PfDyn2 to the mitochondrion in the schizont stage, but not in the ring or trophozoite stages. DAPI stains nucleus. PfDyn2 was detected by α-HA. The mitochondrion was labeled with MitoTracker Red CMXRos (60 nM). Images were taken with a Nikon Ti microscope and processed by Nikon NIS elements software.

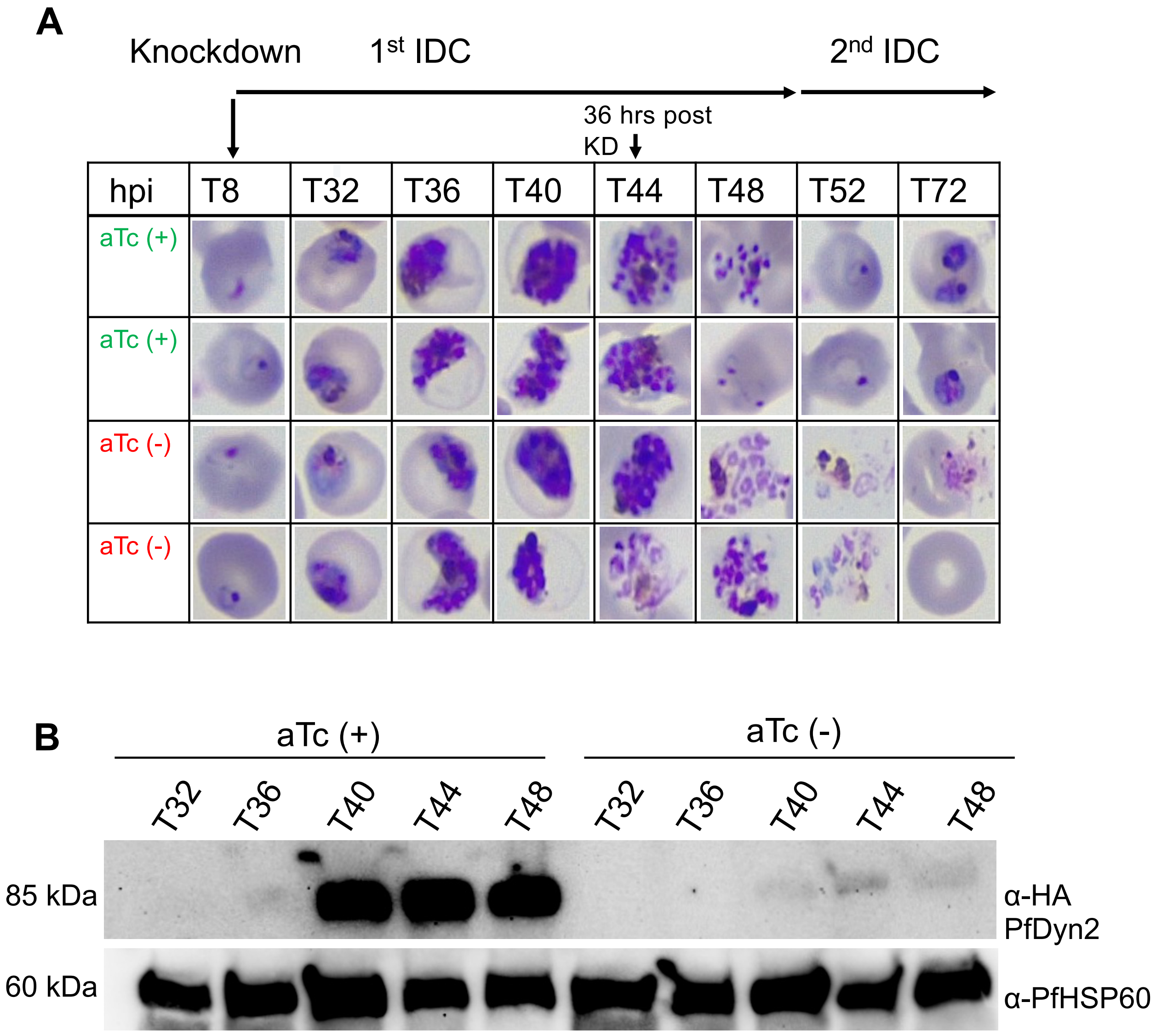


**Supplementary Figure 3**. Field stain and protein quantification of PfDyn2-sufficient and -deficient parasites in a 4-h time course experiment.

**Figure S3. A**) Giemsa-stained images of intraerythrocytic development cycle (IDC) showing the morphologies of PfDyn2 sufficient and deficient parasites. aTc was removed from a tightly synchronized ring stage culture. At 36-h post aTc removal, abnormal morphologies in PfDyn2 deficient parasites were visible. **B**) Western blots comparing PfDyn2 expression levels in PfDyn2-sufficient and -deficient parasites. In the presence of aTc, PfDyn2 was detected in the schizont stage. Upon aTc removal, PfDyn2 levels fell below the detection limit. PfHSP60 was used as a loading control (α-PfHSP60, NBP2-12734, Novus).

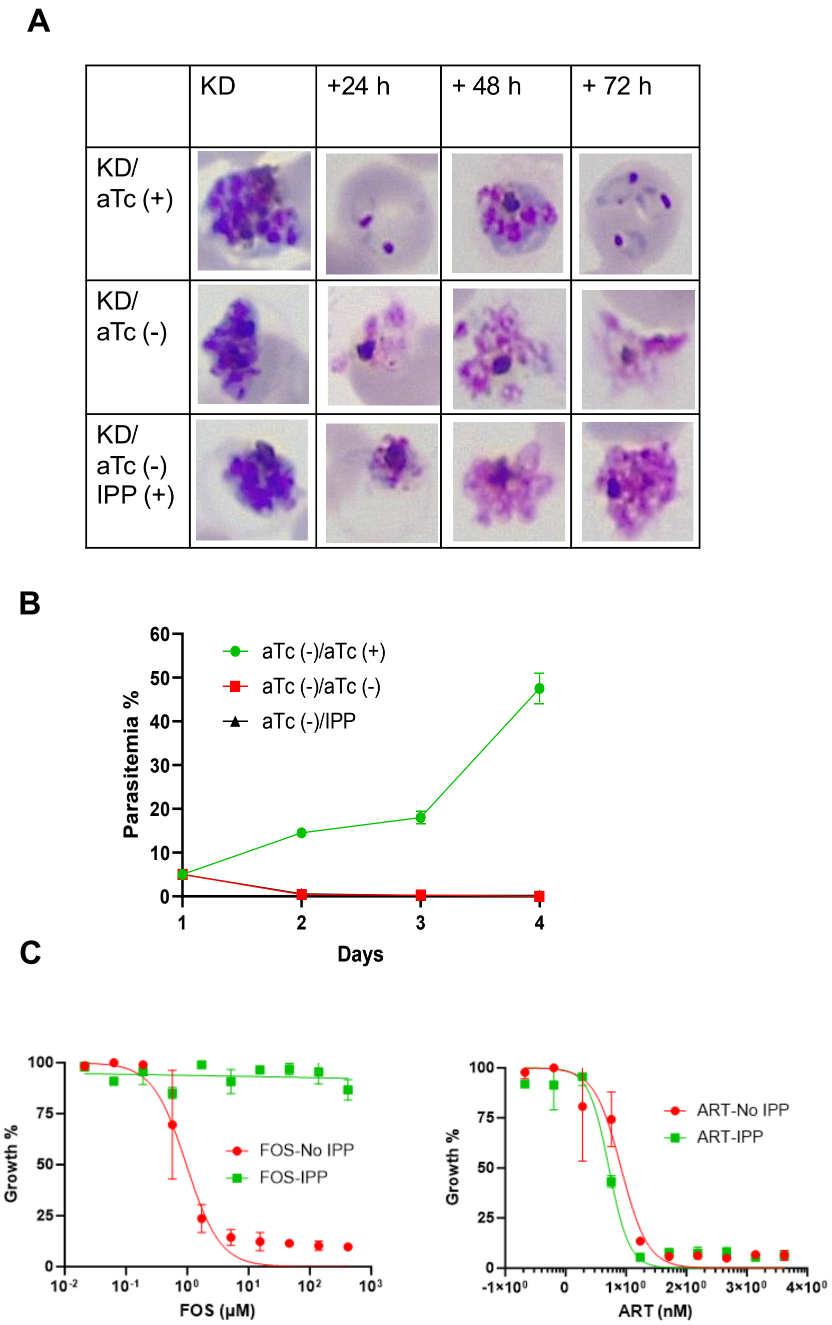


**Figure S4**. PfDyn2 deficient parasites cannot be rescued with IPP.

**Figure S4. A**) Morphologies of PfDyn2-deficient parasites rescued with media supplemented with aTc (+), aTc (-), or aTc (-) + IPP (200 μM, IPP001, Isoprenoids, LC). KD, knockdown (removal of aTc) for 36-h from a tightly synchronized ring stage culture. **B**) Growth curves of PfDyn2 deficient parasites rescued with aTc (+), aTc (-), or aTc (-) + IPP (200 μM). Parasitemia was determined from microscopic counting of at least 1,000 red blood cells in each thin blood smear and is the product of actual parasitemia and splitting factors. Data displayed as mean ± s.d. of three replicates. **C**) Growth inhibition assays showing toxicity of fosmidomycin, but not artemisinin, can be reversed with IPP (200 μM). SYBR green assays were performed according to the standard 72-h protocol starting from ring stage parasites. Data displayed as mean ± s.d. of three replicates.

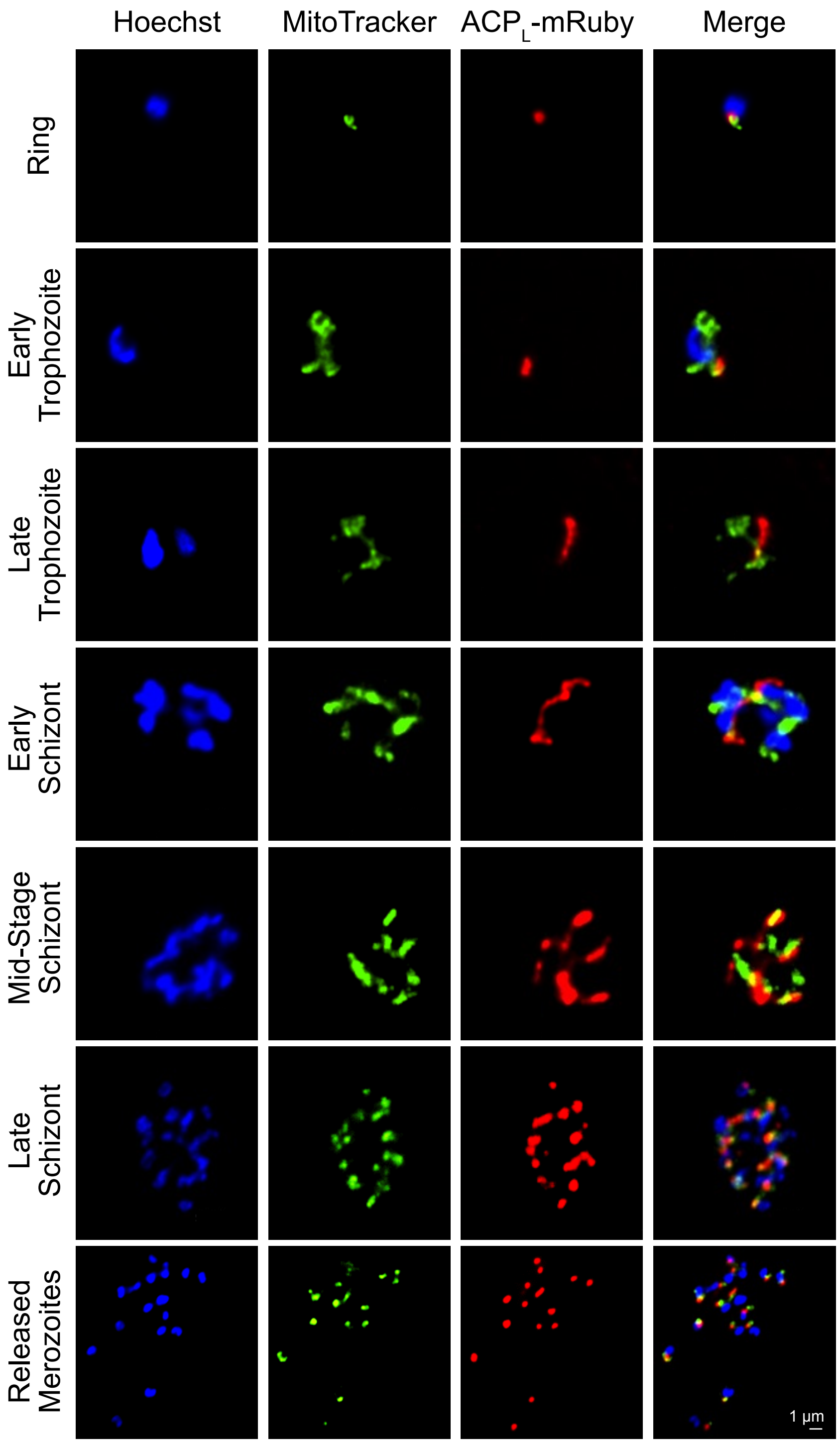


**Supplementary Figure 5.** Morphologies of the apicoplast labeled with ACP$_L$-mRuby.

**Figure S5.** In the PfDyn2-3HA$^{apt}$ line, the apicoplast was labeled with ACP$_L$-mRuby and the mitochondria were was labeled with MitoTracker Green FM (M46750, ThermoFisher). Images of live cells were taken on a Nikon Ti microscope and processed by Nikon NIS elements software.

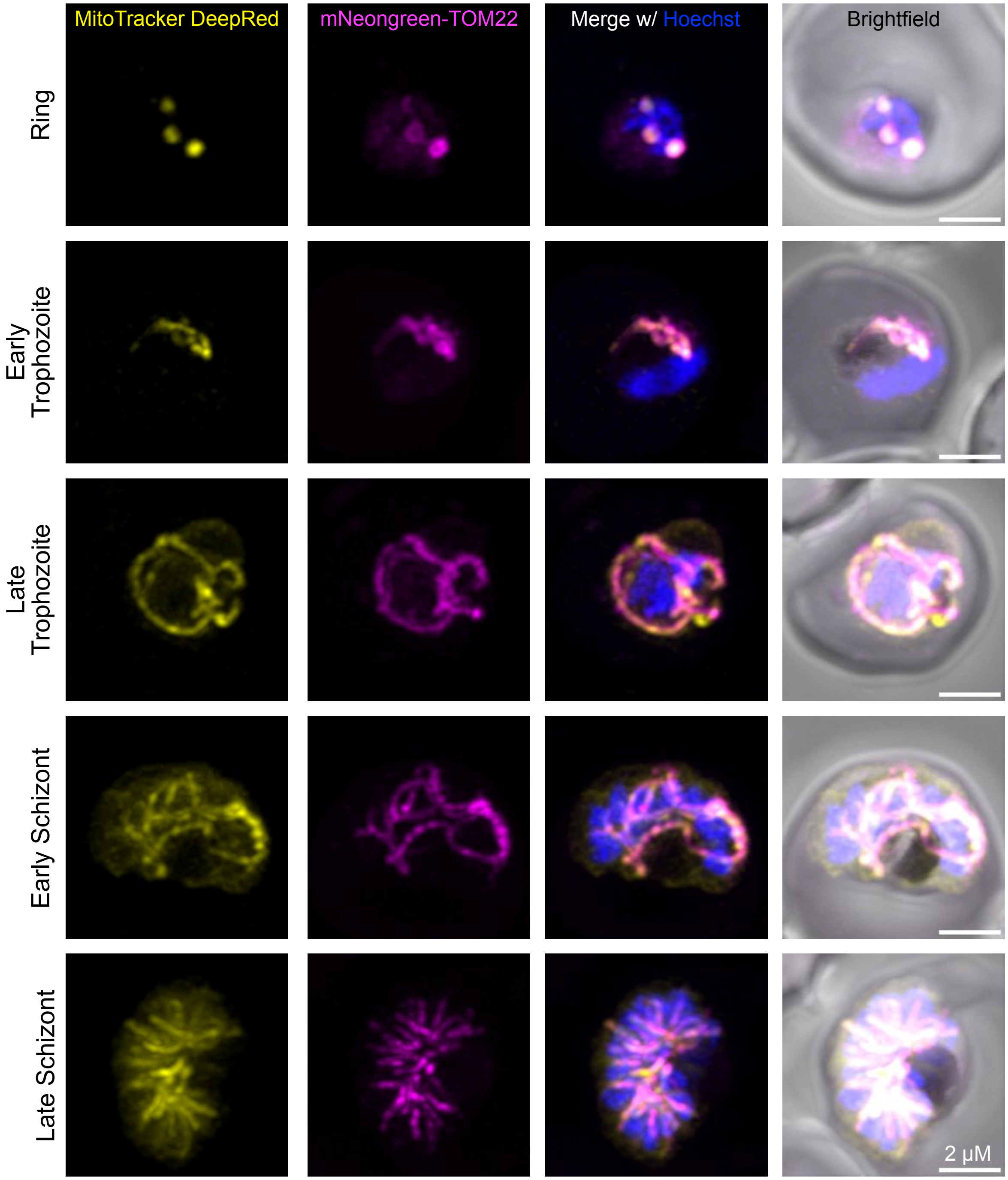


**Figure S6**. Morphologies of mitochondria labeled with mNeonGreen-Tom22.

**Figure S6.** In the PfDyn2-3HA$^{apt}$ line, the mitochondrion was labeled with mNeonGreen-Tom22 and stained with MitoTracker Deep Red FM. Images of live cells were taken on LSM900 with Airyscan2 and processed with FIJI (shown as maximum projections).

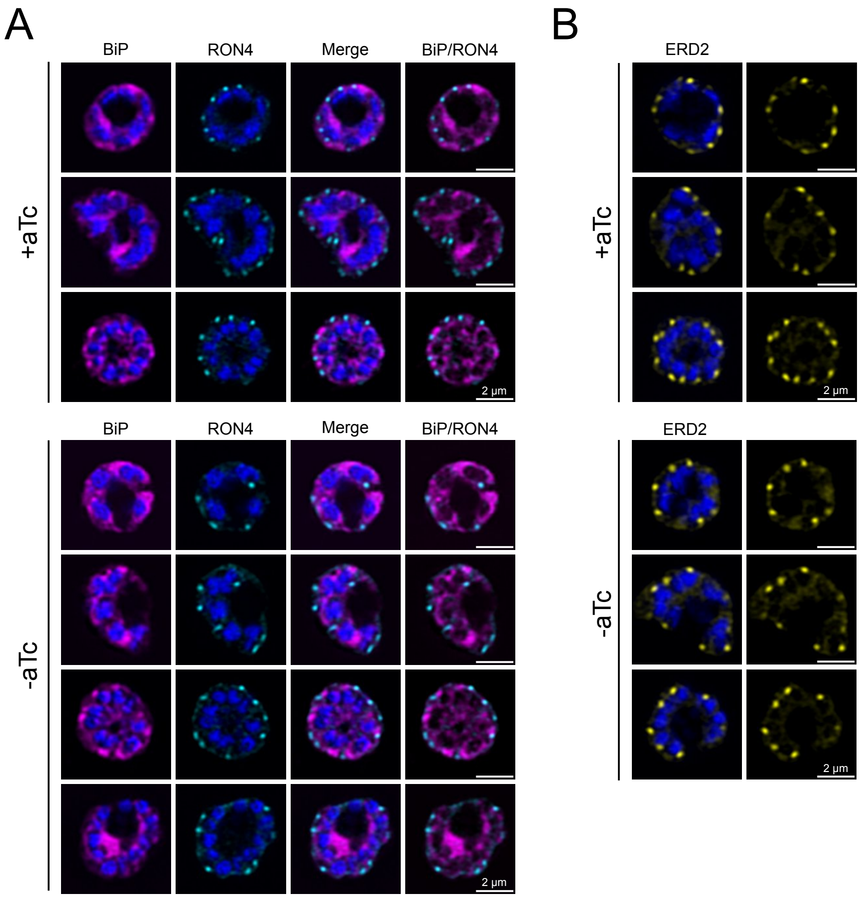


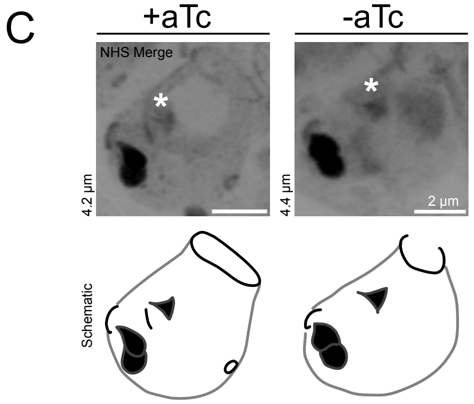


| Condition | Forming Merozoites with 1:1 ratio of CP to APR |
|---|---|
| +aTc | 59 of 59 (100%) |
| -aTc | 49 of 49 (100%) |

**Figure S7**. Evaluation of rhoptries, endoplasmic reticulum, Golgi, and centriolar plaques in PfDyn2-sufficient and PfDyn2-deficient schizonts.

**Figure S7.** In the PfDyn2-3HA$^{apt}$ line, parasites were grown with (+aTc) and without (-aTc) from the ring stage until fixation as schizonts. Images of fixed cells stained with antibodies against **A)** PfRON4 (rhoptry marker, green) and PfBiP (endoplasmic reticulum, magenta) or **B)** PfERD2 (Golgi, yellow) were taken on LSM900 with Airyscan2 and processed with FIJI (single slices shown). **C)** PfDyn2-sufficient and -deficient parasites were evaluated by U-ExM and stained with AlexaFluor 405-NHS ester. The apical polar rings (APR) were identified and counted, then the centriolar plaques (CP, indicated with white asterisks) were enumerated. The table on the right shows the counts from 59 nascent merozoites from two different +aTc parasites and 49 nascent merozoites from two different -aTc parasites. An example image from a +aTc and -aTc parasite are shown with a schematic below. Images were obtained on LSM900 with Airyscan2 and processed with FIJI (shown as maximum projections).

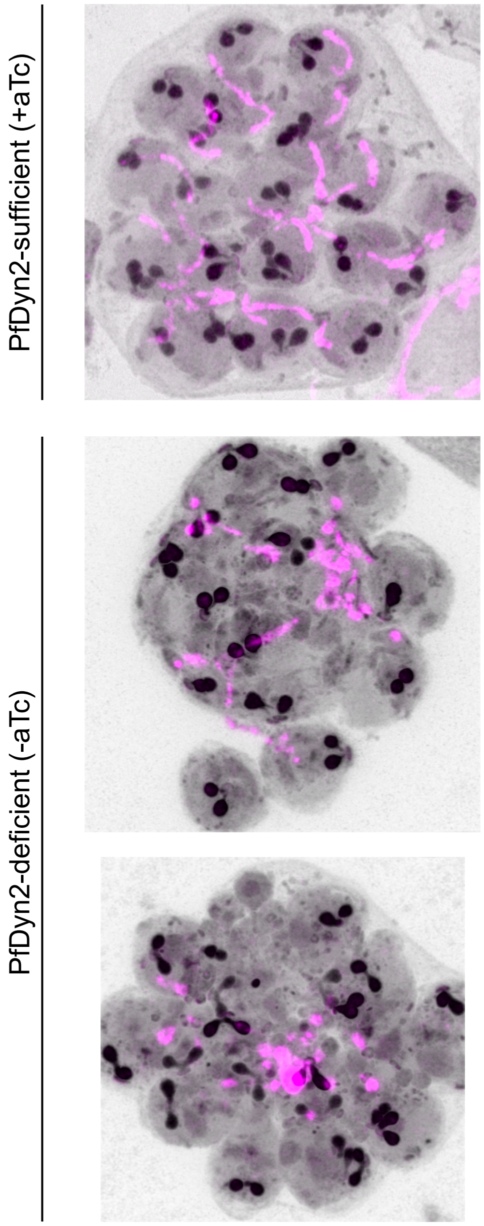


**Figure S8.** Maximum projections of entire schizonts from Figure 4A.

**Figure S8.** Maximum projections of entire schizonts from Figure 4A. The maximum projections show the AlexaFluor 405-NHS-ester (gray) and MitoTracker (magenta) for the full schizonts. Images were obtained on LSM900 with Airyscan2 and processed with FIJI.

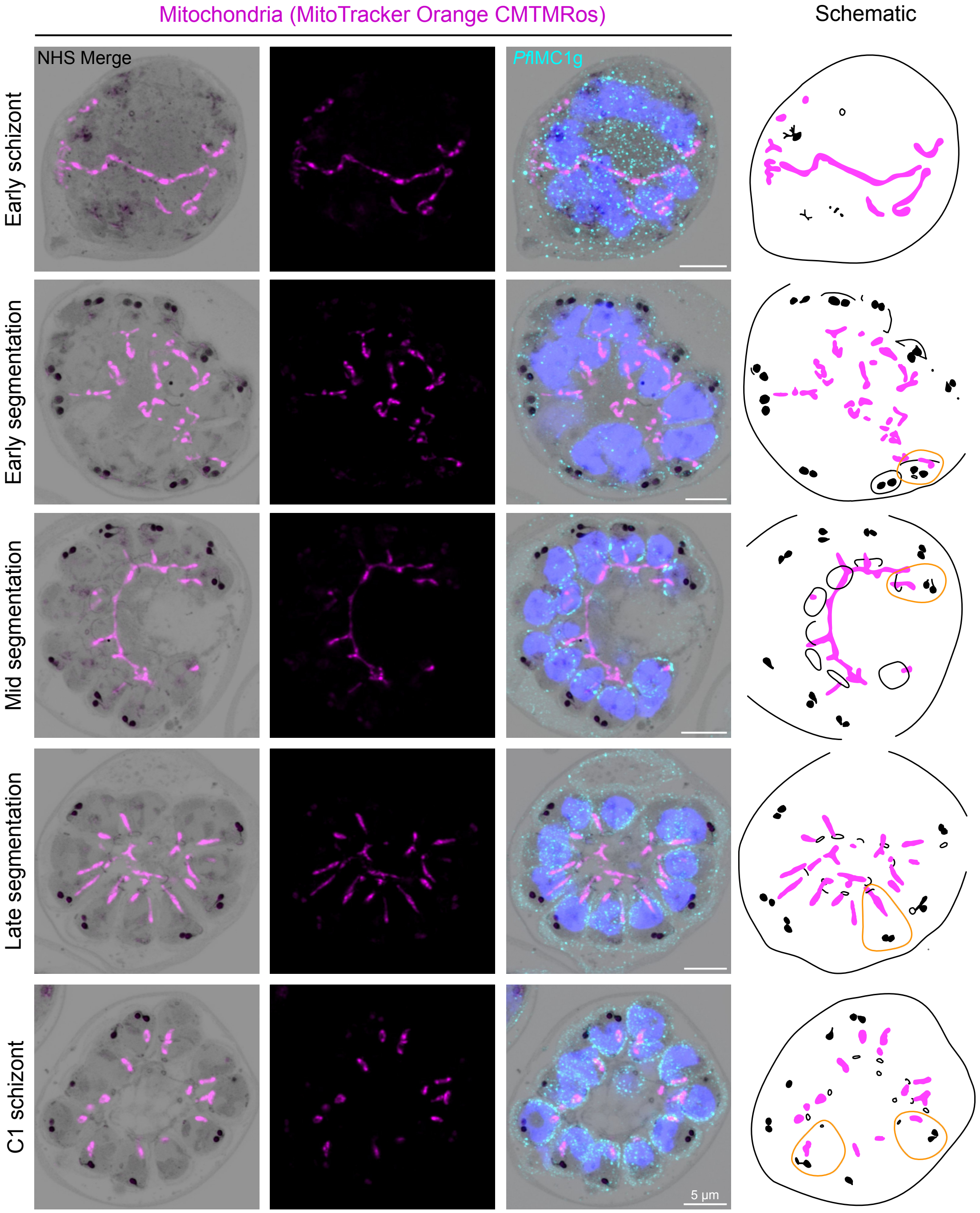


**Figure S9**. Fixed-cell time course of IMC formation and mitochondrial fission.

**Figure S9.** In the PfDyn2-3HA$^{apt}$ line (+aTc), the mitochondria were stained with MitoTracker Orange CMTMRos (magenta) prior to fixation and preparation for U-ExM. The IMC was stained with anti-PfIMC1g (cyan). Images were taken on LSM900 with Airyscan2 and processed with FIJI (maximum projection images of a subset of 20 z-slices are shown for each image, expect for the early schizont that shows 35 slices). Schematics of the parasites are shown on the right side.

**Table S1.** Primers and oligomers used in this study.

| ID | NAME | SEQUENCE |
|---|---|---|
| P1 | DYN2-3HRFP | ATGCGCGCTGGGATCTATCTATTTTGGTAATATC |
| P2 | DYN2-3HRRP | TCCGGAATTGATATCCCGCGGAGATTAGTCATGGAATT ATTATAAGA |
| P3 | DYN2-5HRFP | CCGCGGGATATCAATTCCGGAAGTAGTTCACCCTAAAC ATAATC |
| P4 | DYN2-5HRRP | ATGGTAACCAACACCTCCTGATTCCGTATCTCTGCCAAC ATTTTTATTGCTTGATTTAA |
| P5 | pMG75SeqF | CTTTAAATTCATGCAAAAATTTAC |
| P6 | BBHARev | GTCAGGAACGTCGTATGGATA |
| P7 | DYN2-gRNA1 | CATATTAAGTATATAATATTGTATTTATATTTACATCCAT GGTTTCAGAGCTATGCTGGA |
| P8 | DYN2-gRNA2 | CATATTAAGTATATAATATTAACTTCTTGGTTTCTAATTT GTTTCAGAGCTATGCTGGAA |
| P9 | SuperCPF148bp | CTTTTATTTTTACTGTAATATAATTTTTTATAATGTAAAAATA AAGGG |
| P10 | DYN2-5fout | GATAAAGATAATGATTATATAGATG |
| P11 | DYN2-3fout | GAAATTCAATATAAAGATTAGTCATGG |
| P12 | HSP60LF | GTTAATATTATACAATATACCTAGGATGATATCAACATTA CGAGG |
| P13 | HSP60LR | GCTAGCGATAACATTTCTTCCTTTTGG |
| P14 | neonGreenF | GGAAGAAATGTTATCGCTAGCGGAAGTGGAGGAGTGA GCAA |
| P15 | neonGreenR | GAGTACATAAATATATTATATAACTCGACCTTAAGTCACT TGTACAGCTCGTCCA |
| P16 | PLN-RL2-5’Seq | GTAAGTTCATTTTACCAGTTAAG |
| P17 | PLN3’Seq | GTAGACCCCATTGCGAGTAC |
| P18 | TWINSTREPCam | TTTTATAATAATAAATACCTAATAGAAATATATCACCTAG GAAAAATGTGGAGTCATCCACAATTCGAAAAAGGATCT |

| | | |
|---|---|---|
| | | GGAGGAGCAAGTTGGAGTCATCCACAATTCGAAAAAGG AAATGGAAATGCTAGCGGAAGTGGAGGAGTGAGCAAG |
| P19 | STREPtagF | TGGAGTCATCCACAATTCGAAAAAG |
| P20 | PLN-CAM-5'Seq | ATGTATATTTTAAACTAGAAAAGGAATAAC |
| P21 | PfTOM22F | CGTGTACAAGGGATCTGGATCTCGTACGATGGGAACAG CACTATCAAAAATTATTACG |
| P22 | PfTOM22R | GTCTTAAGTTAGTTTAATTGTGGAACATTGGC |
| P23 | mRUBYiATPSnFR1.0F | ATCCTAGGGCTAGCAAAAATGGTGTCTAAGGGCGAAGA GCTG |
| P24 | mRUBYiATPSnFR1.0R | ATCTTAAGTTACCCGGGTTCGAACTTGTACAGCTCGTC CATGC |
| P25 | ACP55aaAVRIIF | ATCCTAGGAAAAATGAAGATCTTATTACTTTGTATAATTT TTC |
| P26 | ACP55aaNHEIR | ATGCTAGCGAACCACTTCCTGGGTTTTTATTTTTTATCA AATTGTAATC |
| P27 | Dyn2-5INT-F | TAAATTGTTTAAAGGAACCTTCACTTCGTTGTG |
| P28 | Dyn2-5INT-R | GTAGTCAGGAACGTCGTAAGGGTAAGAACC |
| P29 | Dyn2-3INT-F | AAATATATATCCAATGGCCCCTTTCCGGG |
| P30 | Dyn2-3INT-R | TGATATTGTTCAGAATATAAACGTATTGACA |

**Supplemental Video Legends:**

Title: Supplemental Video 1

Description: 3-dimensional rendering of time-lapse confocal (AiryScan Multiplex 4Y) imaging of PfDyn2-3HA$^{apt}$-ACP$_L$-mRuby (PfDyn2-sufficient condition). ACP$_L$-mRuby is shown in green alone (right panel) and merged with transmitted light / brightfield illumination of cellular morphology (left panel). Parasites were imaged with Z-stacks every 20 minutes for a total of 13 hours, the selected parasite for video completed segmentation in 5-6 hours; frame rate is 1 second per 20-minute time point. Scale bar = 1 µm. Corresponds with Figure 2a (top rows).

Title: Supplemental Video 2

Description: 3-dimensional rendering of time-lapse confocal (AiryScan Multiplex 4Y) imaging of PfDyn2-3HA$^{apt}$-ACP$_L$-mRuby parasites (PfDyn2-deficient condition). ACP$_L$-mRuby is shown in green alone (right panel) and merged with transmitted light / brightfield illumination of cellular morphology (left panel). Parasites were imaged with Z-stacks every 20 minutes for a total of 13 hours, the selected parasite for the video completed segmentation in 5-6 hours; frame rate is 1 second per 20-minute time point. Scale bar = 1 µm. Corresponds with Figure 2a (bottom rows).

Title: Supplemental Video 3

Description: 3-dimensional rendering of time-lapse confocal (AiryScan Multiplex 4Y) imaging of PfDyn2-3HA$^{apt}$-StrepII-mNeonGreen-Tom22 parasites (PfDyn2-sufficient condition). mNeonGreen-Tom22 is shown in magenta alone (right panel) and merged with transmitted light / brightfield illumination of cellular morphology (left panel). Parasites were imaged with Z-stacks every 20 minutes for a total of 13 hours, the selected parasite for the video completed segmentation in 5-6 hours; frame rate is 1 second per 20-minute time point. Scale bar = 1 µm. Corresponds with Figure 3a (top rows).

Title: Supplemental Video 4

Description: 3-dimensional rendering of time-lapse confocal (AiryScan Multiplex 4Y) imaging of PfDyn2-3HA$^{apt}$-StrepII-mNeonGreen-Tom22 parasites (PfDyn2-deficient condition). mNeonGreen-Tom22 is shown in magenta alone (right panel) and merged with transmitted light / brightfield illumination of cellular morphology (left panel). Parasites were imaged with Z-stacks every 20 minutes for a total of 13 hours, the selected parasite for the video completed segmentation in 5-6 hours; frame rate is 1 second per 20-minute time point. Scale bar = 1 µm. Corresponds with Figure 3a (bottom rows).

Title: Supplemental Video 5

Description: 3-dimensional rendering of time-lapse confocal (AiryScan Multiplex 4Y) imaging of PfDyn2-3HA$^{apt}$-PfCINCH-mNeonGreen parasites (PfDyn2-sufficient condition) stained with 10 nM MitoTracker Deep Red FM. PfCINCH-mNeonGreen (green) and MitoTracker (magenta) are merged in the leftmost panel, PfCINCH alone is shown in the center panel, and MitoTracker alone is shown in the rightmost panel. Parasites were imaged with Z-stacks every 20 minutes for a total of 13 hours, the selected parasite for the video completed segmentation in 8-9 hours; frame rate is 1 second per 20-minute time point. Scale bar = 1 µm. Corresponds with Figure 4b (top rows).

Title: Supplemental Video 6

Description: 3-dimensional rendering of time-lapse confocal (AiryScan Multiplex 4Y) imaging of PfDyn2-3HA$^{apt}$-PfCINCH-mNeonGreen parasites (PfDyn2-deficient condition) stained with 10 nM MitoTracker Deep Red FM. PfCINCH-mNeonGreen (green) and MitoTracker (magenta) are merged in the leftmost panel, PfCINCH alone is shown in the center panel, and MitoTracker alone is shown in the rightmost panel. Parasites were imaged with Z-stacks every 20 minutes for a total of 13 hours, the selected parasite for the video completed segmentation in 11 hours; frame rate is 1 second per 20-minute time point. Scale bar = 1 µm. Corresponds with Figure 4b (bottom rows).
